# Current Situation, Global Potential Distribution and Evolution of Six Almond Species in China

**DOI:** 10.3389/fpls.2021.619883

**Published:** 2021-04-23

**Authors:** Wei Wang, Zhen-Jian Li, Ying-Long Zhang, Xin-Qiao Xu

**Affiliations:** ^1^Key Laboratory of Silviculture of the State Forestry Administration, The Institute of Forestry, The Chinese Academy of Forestry, Beijing, China; ^2^Shenmu County Association of Ecological Protection and Construction, Shenmu, China

**Keywords:** almond species, current situation, evolution, MAXENT model, potential distribution, climate variable

## Abstract

Almond resources are widely distributed in Central Asia; its distribution has not been studied in detail. Based on the first-hand data of field investigation, climate variables and chloroplast genome data, climatic characteristics of six almond species in China were analyzed, and the global distribution and evolutionary relationship were predicted. The six almond species are concentrated between 27.99°N and 60.47°N. Different almond species have different climatic characteristics. The climate of the almond species distribution has its characteristics, and the distribution of almond species was consistent with the fatty acid cluster analysis. All the test AUC (area under curve) values of MaxEnt model were larger than 0.92. The seven continents except for Antarctica contain suitable areas for the six almond species, and such areas account for approximately 8.08% of the total area of these six continents. Based on the analysis of chloroplast DNA and the distribution characteristics, the evolutionary relationship of the six almond species was proposed, which indicated that China was not the origin of almond. In this study, the construction of a phylogenetic tree based on the chloroplast genome and the characteristics of geographical distribution were constructed. The six almond species in China may have evolved from “Unknown almond species” through two routes. The MaxEnt model for each almond species provided satisfactory results. The prediction results can provide the important reference for *Prunus dulcis* cultivation, wild almond species development and protection.

## Introduction

Fruit species are adding value of earth’s diversity and fundamental to all life. Research into the temperate fruit crops requires the integration of both basic and applied aspects of plant physiology, ecology and genetics (Eyduran et al., 2015; Olak et al., 2019; Životić et al., 2019). Almond [*Prunus dulcis* (Mill.) D.A. Webb] is one of the most important tree nut crop in terms of commercial production. In addition to the almond cultivars, there are more than 30 species of wild almond around the world, five wild almond resources have been reported in China (Wang et al., 2019, 2020), including the high-latitude species of wild almond (*Prunus tenella* Batsch), the desert and mountain species of longstalk almond [*Prunus pedunculata* (Pall.)], the high and low temperature suitable species of flowering almond (*Prunus triloba* Lindl.), the high altitude species Tangut almond [*Prunus tangutica* (Batalin) Koehne], and the northern mountain species Mongolic almond (*Prunus mongolica* Maxim.). All wild almond species are highly adapted to cold and dry climates, which may be a commercially important gene pool. At present, wild almond genetic resources have attracted much attention for their nut chemical compositions and possible uses (Sorkheh et al., 2016; Wang et al., 2018b, 2019). Furthermore, it is of great significance to investigate the almond geographical and climatic characteristics, and to evaluate their potential distribution areas and their evolutionary relationships for effective utilization and preservation.

Previous studies have mainly focused on abiotic stress (Ighbareyeh et al., 2018; Kodad et al., 2018), chemical composition (Čolić et al., 2017; Wang et al., 2019), genetic diversity (Sorkheh et al., 2017), and variety classification (Xie et al., 2006) of almond resources, while little is known about the distribution of their habitat and the ecological requirements affecting their sustainability. Therefore, detailed habitat assessments, especially the assessment of climate factors such as temperature and rainfall, are the most important step for guiding the improvement of almond production, and are also necessary basic research on the exploitation and utilization of plant resources.

Almond yield and chemical composition are closely related to climate factors. Almond production depends on the climate factors such as precipitation, mean and extreme temperatures and soil water reserves, and bioclimate factors such as water deficits (Ighbareyeh et al., 2018). The tocopherol concentration in almond depends on the genotype and the environment, such as the climatic conditions around the year and the growing management practices of the orchard (Kodad et al., 2018). The almond lipid content and fatty acid composition are dependent not only on the genotype but also the location and climatic conditions prevalent during the growing season (Sathe et al., 2008). All of these studies have been shown confirmed that climate plays an important and active role in physiology, plant productivity, and other plant processes. Exploring the correlation between the almond species and environmental variables, along with determining the factors influencing its distribution, are of great significance. This should be done to gain an understanding of the scope of the area suitable for the cultivation of almond species and to guide almond species cultivation.

Climate plays a decisive role in species distribution and serves as the most important factor affecting biological growth and reproduction (Zhang et al., 2019; Li et al., 2020). When the data on a species’ distribution are limited, species distribution models can be used to determine the ecological needs of the species and to estimate its potential range with respect to regional ecology and biogeography. At present, the species distribution models mainly include the bioclimatic prediction system (Bioclim) (Busby, 1991), the domain model (Carpenter et al., 1993), ecological niche factor analysis (ENFA) (Hirzel and Guisan, 2002), maximum entropy models (MaxEnt) (Phillips et al., 2006), and genetic algorithm for rule-set prediction (GARP) (Stockwell and Peters, 1999). Among these models, the MaxEnt model simulates the geographical distribution of species. Compared with the other models, the MaxEnt model has the advantages of easy operation, small sample size requirements, short model running time, incomplete datasets, and high simulation precision (Tsoar et al., 2007; Srivastava et al., 2018). MaxEnt uses a grid format to express how different environmental variables affect the suitability of a species habitat. If a specific grid point is specified as appropriate, this means that the grid point has the most suitable climatic and environmental conditions for a certain species (Phillips et al., 2006). MaxEnt is a preferred method compared to other current methods because it deals only with “data present”; it is difficult to detect and collect absent data, and such data are rarely available (Pearson et al., 2007; Phillips and Dudík, 2008). The output of the MaxEnt model is continuous, rather than deterministic (e.g., GARP) (Stockwell and Peters, 1999; Wang et al., 2017).

In this work, the climate suitability of six almond species in China was analyzed for the first time by MaxEnt and ArcGIS. The main climatic factors and suitable growing areas were determined, which provides a scientific basis for the introduction and cultivation of almond species in the future.

In this study, the six almond species were the focus of analysis, including *P. dulcis*, *P. mongolica*, *P. pedunculata*, *P. tangutica*, *P. tenella*, and *P. triloba*. The MaxEnt model was used to analyze the potential distribution areas of the six almond species. This analysis would help establish which environmental factors affect habitat distribution. The chloroplast genome and the fatty acid composition were used to determine the relationships of the six almonds. This study was the first to combine the characteristics of species geographic distribution with the evolution of the chloroplast genome and an analysis of chemical composition. The results provide a reasonable basis for the assessment of habitat suitability and resource conservation for the protection of wild almonds.

According to the georeferenced occurrence records of the six almond species and high-resolution environmental data, information technologies, such as the MaxEnt model and ArcGIS technologies, were applied to evaluate the current spatiotemporal distribution and potential habitat of the six almond species in China. The aims of this study were to (1) obtain the current global spatiotemporal distribution information of six almond species; (2) determine the important environmental variables that are highly correlated with the potential distribution range of the six almond species; (3) predict the potential global distribution; and (4) construct a phylogenetic analysis based on the chloroplast genome of the six almond species. The results will provide theoretical support and a reference for the cultivation and promotion of almond resources.

## Materials and Methods

### Collection of Data on the Distribution Points of Almond Species in China and Other Countries

The almond resource distribution point data were collected from two sources. The data for almond resources in China were obtained from field surveys from 2012 to 2019. The surveys covered northern, northwestern, and southwestern China. The data for almond resources in other parts of the world were collected from websites [the Global Biodiversity Information Facility (GBIF)^1^ ]. ArcGIS software (version 10.2, ESRI, Redlands, CA, United States) was selected for the analysis of the actual distribution, richness, and diversity of the almond species. The diversity, geographical distribution, and richness recorded on germplasm accessions were mapped using ArcGIS to produce a 1° × 1° decimal degree size grid map.

### Environmental Parameters

In this study, 26 variables related to the distribution of six almond species were selected, including 19 variables representing bioclimatic factors (bio01-bio19), 3 topographic variables (Elev, Aspect, and Slope), and 4 soil variables (T_OC, T_PH_H2O, T_SAND, and T_SILT). The climate data include 19 bioclimatic variables and elevation variables from the WorldClim Version 2 dataset at 30 arc-second resolution^2^ (Fick and Hijmans, 2017). Topographic data, including elevation (Elev), slope (Slope), and aspect (Aspect) were extracted using ArcGIS 10.2. The soil data are from the Harmonized World Soil Database v1.2^3^ and include 30 arc-second resolution rasters for topsoil organic carbon, pH, percent silt, and percent sand (Fischer et al., 2008).

These environmental variables are listed in Table 1. With the help of ArcGIS Conversion Tools, the environmental factors were converted into ASCII format. All environmental data were projected to USA Contiguous Albers Equal Area Conic (NAD 1983) and resampled using nearest-neighbor to a 30 arc-second resolution by ArcGIS.

**TABLE 1:** Global spatial distribution of sample points for the six almond species.

Latin name of species	Sampling distribution	Sampling points	Investigation points	GBIF website points
*P. pedunculata*	Global: China, Mongolia China: Inner Mongolia, Shaanxi	459	433	26
*P. triloba*	Global: China, United States, Germany, France, etc. China: Hebei, Shaanxi, Inner Mongolia, etc.	153	59	94
*P. mongolica*	Global: China, Mongolia China: Inner Mongolia, Ningxia	96	96	0
*P. tangutica*	China: Sichuan, Gansu, etc.	166	166	0
*P. tenella*	Global: China, Russia, Kazakhstan, Sweden, Ukraine, etc. China: Xinjiang	493	178	315
*P. dulcis*	Global: China, Spain, Portugal, United States, etc. China: Xinjiang	242	21	221
Total		1609	853	656

### Prediction of the Almond Species Potential Distribution by MaxEnt

The maximum entropy algorithm (MaxEnt 3.3.3k) model (Phillips et al., 2006) was applied to predict the potential distribution area of the six almond species. The 26 variables and the species occurrence data were loaded into the MaxEnt model; 75% of the location data were used as training data, and the remaining 25% were randomly set aside as test points and used to compute the area under the curve (AUC), omission rate, and other parameters. At the same time, the jackknife method was applied to assess the relative importance of the variables. The suitability maps were calculated using the logistic output of MaxEnt, which ranges from 0 to 1. For visualization and further analysis, the MaxEnt results were imported into ArcGIS 10.2 and the habitat suitability maps were divided into four levels: high habitat suitability (>0.66), moderate habitat suitability (0.33–0.66), low habitat suitability (0.05–0.33), and unsuitable habitat (<0.05).

### Cluster Analysis of Fatty Acids

The fatty acid data of five wild almonds are from our previous reports (Supplementary Table 1) (Wang et al., 2018b, 2019). Combined with the fatty acid composition of *P. dulcis*, a dendrogram (cluster) was created to show the relationships among the investigated plant samples by SPSS 18.0. The fatty acid composition includes total saturated fatty acids (SFAs), monounsaturated fatty acids (MUFAs), and polyunsaturated fatty acids (PUFAs). The trigonometric diagram was drawn by Sigmaplot 10.0. The three angular points in the triangular graph represent 100% of MUFAs, PUFAs, and SFAs.

### Phylogenomic Analysis

To reveal the evolutionary relationship among the six almond species, the complete chloroplast genomes of the six almond species were collected from GenBank, which are the chloroplast genome sequences assembled by our team (Wang et al., 2018a, 2020). The phylogenetic trees based on maximum likelihood analysis were constructed by PhyML v3.0^3^, and the bootstrap repetition rate was 1000.

### Statistical Analysis

The data of this study were analyzed by SPSS 18.0 software. Data for all measurements were expressed as the mean values, and Tukey’s test was used to detect significant differences (*p* < 0.05) between these values. Principal component analysis (PCA) was applied to observe any possible clusters within the analyzed climate data of the six almond species using PAST3 software. MATLAB 8.3.0.220 software and SigmaPlot 10.0 software were used for data graph processing.

## Results

### Analysis of the Current Almond Species Distribution

**FIGURE 1:**
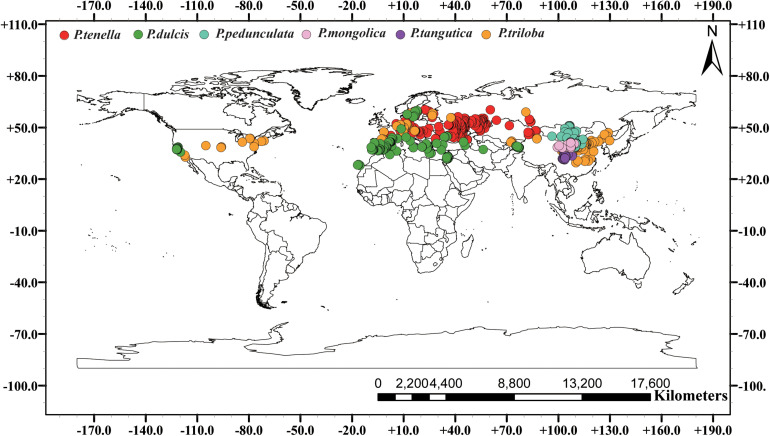
Global spatial distribution of sample points for the six almond species, (red) *P. tenella*; (green) *P. dulcis*; (light blue) *P. pedunculata*; (pink) *P. mongolica*; (purple) *P. tangutica*; (brown) *P. triloba*.

We conducted a field survey in mainland China for 8 years, recording and referring to almost all natural species of subgenus almond. A total of 853 individual sample points were recorded. Among them, 443, 59, 96, 166, 178, and 21 records of *P. pedunculata*, *P. triloba*, *P. mongolica*, *P. tangutica*, *P. tenella*, and *P. dulcis* were collected. Additionally, 26 occurrences for *P. pedunculata*, 94 occurrences for *P. triloba*, 315 occurrences for *P. tenella*, and 221 occurrences for *P. dulcis* were collected from GBIF^4^. The geographic data of species outside the Chinese mainland are from the GBIF website. Including 656 sample points of GBIF, 1609 almond resource points were collected in this study (Table 1 and Figure 1).

#### Analysis of the Geographical Factors (Longitude, Latitude, Altitude, Slope, Aspect)

**FIGURE 2:**
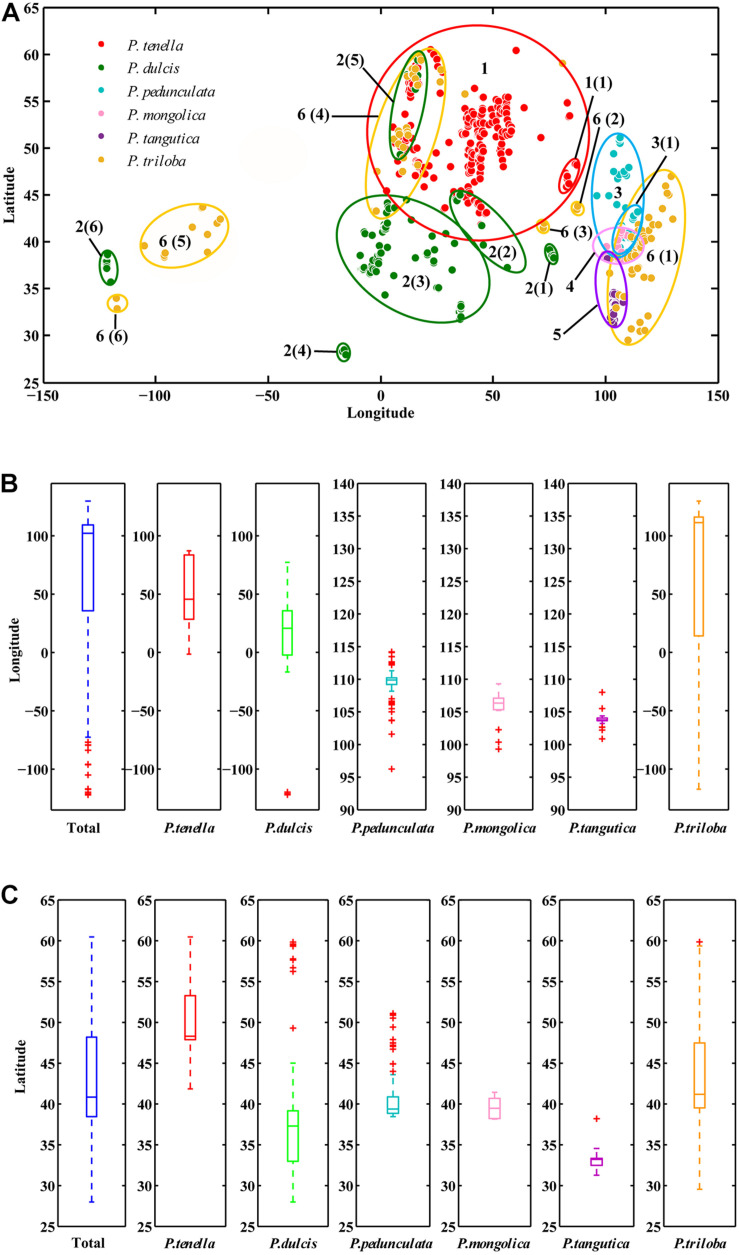
Scatter diagram of longitude and latitude of the six almond species **(A)**; Boxplot of longitude **(B)** and latitude **(C)** of the six almond species by MATLAB.

ArcGIS was used to extract the ecological factor data of the collected sampling points. The sampling points of the six almond species are distributed in middle-latitude regions, 27.99°N-60.47°N latitude (Figures 2A,B and Table 2). The average latitudes of *P. pedunculata*, *P. triloba* and *P. tenella* are above 40°, while those of *P. mongolica*, *P. tangutica*, and *P. dulcis* are below 40°. The distribution spans of *P. triloba*, *P. tenella* and *P. dulcis* in longitude were large. However, the spans of *P. pedunculata*, *P. mongolica*, and *P. tangutica* were small, so their resource distribution was relatively concentrated (Figures 2A,C and Table 2).

**TABLE 2:** Geographic information of the six almond species.

	*Total*	*P. pedunculata*	*P. triloba*	*P. mongolica*	*P. tangutica*	*P. tenella*	*P. dulcis*
Longitude	–122.08 to 129.79 (71.88 ± 1.23)	96.25–114.20 (109.84 ± 0.07)	–117.33 to 129.79 (67.26 ± 5.44)	99.30–109.30 (106.13 ± 0.14)	100.86–107.99 (103.85 ± 0.04)	–1.53 to 87.14 (52.57 ± 1.27)	–122.08 to 77.32 (6.67 ± 3.48)
Latitude	27.99–60.47 (42.58 ± 0.18)	38.45–51.09 (40.26 ± 0.11)	29.54–59.87 (43.56 ± 0.60)	38.15–41.43 (39.66 ± 0.13)	31.27–38.20 (32.94 ± 0.06)	41.86–60.47 (50.28 ± 0.19)	27.99–59.85 (38.42 ± 0.47)
Altitude(m)	5.00–4878.00 (1000.85 ± 19.10)	546.00–3452.00 (1392.08 ± 13.27)	5.00–2438.00 (608.75 ± 47.02)	1073.00–3573.00 (1450.59 ± 36.10)	939.00–4878.00 (2366.58 ± 47.03)	10.00–1710.00 (490.57 ± 20.47)	10.00–2644.00 (430.97 ± 29.67)

**FIGURE 3:**
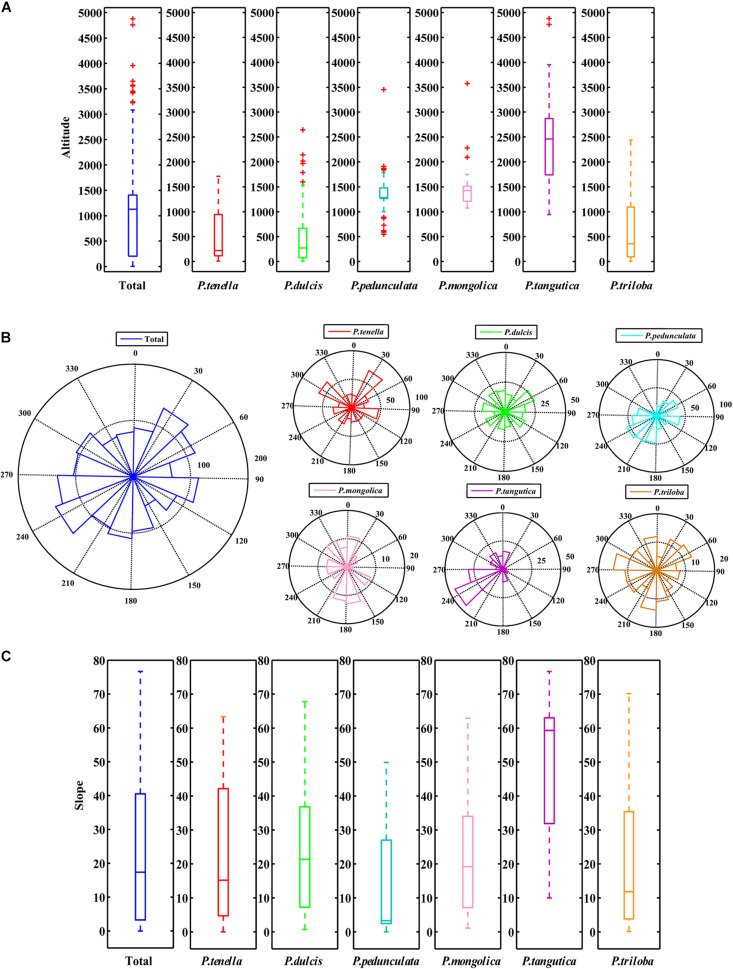
Boxplot of altitude **(A)** and slope **(C)** of the six almond species by MATLAB; Rose diagrams of aspect of the six almond species **(B)**.

There were *P. pedunculata*, *P. mongolica*, and *P. tangutica* at distribution points with an average altitude of more than 1000 m. The average altitude of *P. tangutica* was 2366.58 m (Figure 3A and Table 2). The distribution of almond resources is distributed in each aspect, and the distribution in the positive and negative aspects is relatively less. Among them, *P. mongolica* was more distributed on the positive aspect, *P. tangutica* was mainly distributed on the semipositive aspect, and *P. pedunculata* was more distributed on the positive aspect and semipositive aspect (Figure 3B). The slopes of 75% of the surveyed almond resources were in the range of 0–40°. The mean value of the slope data was lower than 10°; only *P. pedunculata*, *P. triloba*, *P. mongolica*, *P. tenella*, and *P. dulcis* were at 10-30°, while *P. tangutica* was above 30°, with an average slope of 50.49° and a median of 59.60° (Figure 3C).

#### Analysis of the 19 Bioclimatic Parameters (Bio1, Bio5, Bio6, Bio7, Bio12, etc.)

**FIGURE 4:**
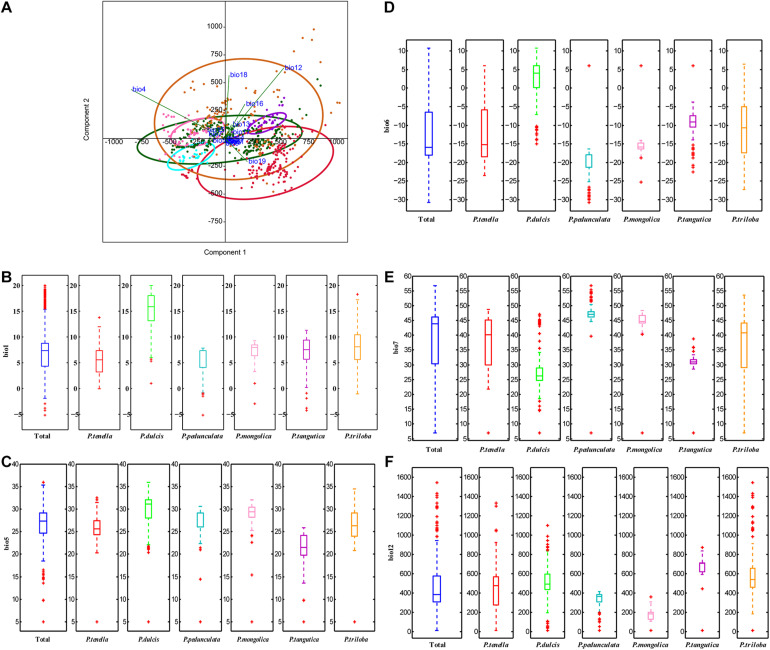
Principal components analysis of the environmental variables **(A)**; boxplot of ecological factor (**B**, Bio1; **C**, Bio5; **D**, Bio6; **E**, Bio7; **F**, Bio12) of the six almond species by MATLAB.

Principal component analysis (PCA) was applied to observe anypossible clusters within the analyzed climate data of the six almondspecies. The scores of the first two principal components of the sixalmond species are shown in Figure 4A and Supplementary Figure 1A. The first twoprincipal components accounted for 90.2% (PC1 = 73.3% andPC2 = 16.9%) of the total variation. PC1 was highly contributed byBio4, Bio12, Bio16, and Bio19 (Supplementary Figure 1B). PC2 was mainly positively correlated with Bio12, Bio18, and Bio4 (Supplementary Figure 1C). Theseresults reinforce the relevance of Bio4, Bio12, Bio16, and Bio19 asdiscriminant parameters to distinguish the climate characteristics of the distribution areas of the six almond species. Among the six almond species, the annual mean temperature of *P. dulcis* was 15.08°C (Tables 3, 4 and Figure 4B). The annual mean temperature of the other five species was relatively low, ranging from 5.53 to 8.22°C (Tables 3, 4 and Figure 4B). The maximum temperature of the warmest month (Bio5) occurred in July (Supplementary Table 2), and the highest temperature of *P. dulcis* was 29.8°C (Supplementary Table 3 and Figure 4C). The minimum temperature of the coldest month (Bio6) occurred in January, among which the average lowest temperature of *P. dulcis* was the highest, 2.10°C. The lowest average temperature of *P. pedunculata* was –19.5°C (Supplementary Table 4 and Figure 4D). The minimum temperatures of the coldest month (Bio6) of *P. triloba*, *P. mongolica*, *P. tangutica*, and *P. tenella* were –11.0, –16.1, –9.4, and –12.6°C, respectively (Supplementary Table 4). Other climatic factors such as Bio2, Bio3, Bio4, and Bio7 are shown in Table 4 and Figure 4E.

**TABLE 3:** Bioclimatic parameters used for the generation of the models of potential distribution of the six almond species.

Field	Description	Units
Bio1	Annual Mean Temperature	°C
Bio2	Mean Diurnal Range (Mean of monthly (max temp - min temp))	°C
Bio3	Isothermality(BIO2/BIO7) (* 100)	°C
Bio4	Temperature Seasonality(standard deviation *100)	%
Bio5	Max Temperature of Warmest Month	°C
Bio6	Min Temperature of Coldest Month	°C
Bio7	Temperature Annual Range(BIO5-BIO6)	°C
Bio8	Mean Temperature of Wettest Quarter	°C
Bio9	Mean Temperature of Driest Quarter	°C
Bio10	Mean Temperature of Warmest Quarter	°C
Bio11	Mean Temperature of Coldest Quarter	°C
Bio12	Annual Precipitation	mm
Bio13	Precipitation of Wettest Month	mm
Bio14	Precipitation of Driest Month	mm
Bio15	Precipitation Seasonality (Coefficient of Variation)	%
Bio16	Precipitation of Wettest Quarter	mm
Bio17	Precipitation of Driest Quarter	mm
Bio18	Precipitation of Warmest Quarter	mm
Bio19	Precipitation of Coldest Quarter	mm
Aspect	Aspect	Degree
Elev	Altitude	m
Slope	Slope	%
T_OC	Topsoil Organic Carbon	% weight
T_PH_H2O	Topsoil pH (H2O)	–log(H +)
T_SAND	Topsoil Sand Fraction	% wt.
T_SILT	Topsoil Silt Fraction	% wt.

**TABLE 4:** Bioclimatic profiles of the six almond species based on the 19 parameters shown in Table 3.

	*P. pedunculata*	*P. triloba*	*P. mongolica*	*P. tangutica*	*P. tenella*	*P. dulcis*
Bio1	–5.18 to 7.82 (5.57 ± 0.11)	–1.08 to 18.27 (8.22 ± 0.29)	–2.99 to 9.27 (7.22 ± 0.19)	–4.24 to 11.32 (7.20 ± 0.20)	–0.05 to 13.76 (5.53 ± 0.13)	5.28–20.01 (15.08 ± 0.24)
Bio2	9.9–15.6 (13.7 ± 0.0)	6.8–16.3 (11.0 ± 0.2)	11.3–15.8 (13.1 ± 0.1)	8.5–14.5 (10.9 ± 0.0)	6.2–12.9 (9.8 ± 0.1)	5.3–15.8 (10.8 ± 0.1)
Bio3	23.6–31.3 (28.9 ± 0.1)	19.6–54.6 (29.9 ± 0.4)	25.4–36.4 (29.2 ± 0.3)	28.5–44.9 (35.1 ± 0.2)	17.0–38.5 (26.1 ± 0.1)	24.8–55.7 (39.4 ± 0.5)
Bio4	1111.0–1608.9 (1230.3 ± 4.0)	395.5–1526.9 (991.2 ± 20.3)	943.3–1246.0 (1172.9 ± 6.8)	633.1–937.6 (712.7 ± 3.7)	503.8–1403.1 (1071.3 ± 11.1)	274.9–1167.5 (643.6 ± 11.5)
Bio5	14.5–30.6 (27.7 ± 0.1)	20.8–34.5 (26.5 ± 0.3)	15.4–32.1 (28.8 ± 0.3)	9.7–25.9 (21.8 ± 0.2)	20.3–32.5 (25.6 ± 0.1)	20.4–35.9 (29.8 ± 0.2)
Bio6	–30.7 to –16.4 (–19.5 ± 0.1)	–27.3 to 6.4 (–11.0 ± 0.6)	–25.3 to –14.0 (–16.1 ± 0.1)	–22.6 to –3.8 (–9.4 ± 0.2)	–23.5 to 2.4 (–12.6 ± 0.3)	–15.0 to 10.7 (2.0 ± 0.4)
Bio7	39.6–56.8 (47.3 ± 0.1)	18.2–53.5 (37.4 ± 0.7)	40.3–48.3 (44.9 ± 0.2)	28.5–38.7 (31.2 ± 0.1)	21.8–48.7 (38.3 ± 0.3)	14.5–47.0 (27.8 ± 0.4)
Bio8	8.5–21.9 (18.5 ± 0.1)	–0.2 to 27.2 (17.9 ± 0.4)	8.2–23.4 (19.8 ± 0.2)	3.8–19.8 (14.6 ± 0.2)	–4.3 to 22.9 (16.5 ± 0.2)	1.9–26.5 (12.6 ± 0.3)
Bio9	–19.8 to –4.8 (–9.9 ± 0.1)	–18.0 to 24.3 (–2.1 ± 0.7)	–13.8 to –4.5 (–7.6 ± 0.2)	–13.9 to 2.3 (–2.1 ± 0.2)	–13.9 to 22.4 (–4.7 ± 0.3)	–6.3 to 26.8 (18.4 ± 0.6)
Bio10	8.5–22.3 (20.0 ± 0.1)	12.8–28.0 (20.0 ± 0.3)	8.2–23.7 (21.1 ± 0.2)	3.8–20.9 (15.6 ± 0.2)	13.6–24.8 (18.4 ± 0.1)	15.3–27.0 (22.8 ± 0.2)
Bio11	–22.0 to –7.3 (–10.4 ± 0.1)	–18.4 to 13.7 (–4.4 ± 0.5)	–15.0 to –5.5 (–7.8 ± 0.2)	–13.9 to 2.3 (–2.1 ± 0.2)	–16.4 to 5.7 (–7.6 ± 0.3)	–8.1 to 13.9 (7.2 ± 0.3)
Bio12	52.0–416.0 (333.7 ± 2.7)	186.0–1541.0 (607.5 ± 19.9)	109.0–360.0 (173.9 ± 5.7)	444.0–873.0 (671.9 ± 4.5)	182–1332 (451.4 ± 8.0)	36.0–1098.0 (497.5 ± 12.3)
Bio13	18.0–108.0 (91.2 ± 0.7)	32.0–287.0 (119.1 ± 3.7)	30.0–91.0 (46.3 ± 1.5)	101.0–173.0 (117.5 ± 0.7)	19.0–193.0 (58.1 ± 0.8)	8.0–208.0 (94.1 ± 2.6)
Bio14	0.0–6.0 (2.6 ± 0.0)	0.0–85.0 (17.3 ± 1.6)	0.0–3.0 (1.0 ± 0.1)	1.0–7.0 (2.1 ± 0.1)	6–85 (22.2 ± 0.6)	0.0–53.0 (8.0 ± 0.8)
Bio15	93.2–123.7 (106.9 ± 0.2)	7.0–139.9 (74.5 ± 3.4)	86.1–110.6 (99.7 ± 0.8)	72.2–104.6 (80.7 ± 0.3)	8.6–54.8 (32.2 ± 0.5)	14.5–117.3 (73.8 ± 2.1)
Bio16	40.0–261.0 (218.6 ± 1.7)	87.0–642.0 (295.5 ± 8.0)	72.0–245.0 (109.3 ± 3.5)	281.0–460.0 (328.1 ± 1.6)	53–492 (155.7 ± 2.4)	17.0–546.0 (247.4 ± 7.0)
Bio17	1.0–24.0 (10.1 ± 0.1)	2.0–261.0 (59.7 ± 5.4)	0.0–10.0 (4.5 ± 0.3)	4.0–28.0 (12.2 ± 0.3)	22–265 (74.2 ± 1.8)	0.0–165.0 (32.0 ± 2.9)
Bio18	40.0–261.0 (210.3 ± 1.5)	11.0–608.0 (278.4 ± 8.7)	72.0–245.0 (106.6 ± 3.3)	281.0–453.0 (320.3 ± 1.7)	45–416 (149.2 ± 2.2)	0.0–220.0 (46.9 ± 4.2)
Bio19	1.0–29.0 (10.1 ± 0.1)	2.0–350.0 (72.5 ± 6.6)	0.0–10.0 (4.5 ± 0.3)	4.0–28.0 (12.2 ± 0.3)	22–456 (89.3 ± 2.3)	1.0–546.0 (225.0 ± 7.8)

The annual precipitation (bio12) was below 400 mm, including 173.9 mm for *P. mongolica* and 333.7 mm for *P. pedunculata*, 451.4 mm for *P. tenella* and 497.5 mm (between 400 and 600 mm) for *P. dulcis* (Figure 4F). The average annual precipitation of *P. triloba* and *P. tangutica* was more than 600, 607.5, and 671.9 mm respectively (Tables 3, 4). In addition to *P. dulcis*, the annual precipitation of the other five almond species was mainly concentrated from June to September in the summer. The annual precipitation of *P. dulcis* was mainly from December to February in the winter (Supplementary Table 5). The water vapor pressure of the six species was mostly lower than 1.00 kPa, showing the characteristics of being high in summer and low in winter. Among them, the water vapor pressure of *P. pedunculata* and *P. mongolic* was the lowest, and only the pressure in June and July exceeded 1 kPa (Supplementary Table 6).

In the distribution area of the six almond species, the solar radiation was relatively strong; only *P. tangutica* was slightly lower (Supplementary Table 7). The average wind speed of *P. mongolica* was the highest in May, which is 3.876 m/s; followed by *P. pedunculata*, *P. triloba*, and *P. tenella*, which were 3.854, 3.553, and 3.490 m/s in April; the highest average wind speed of *P. dulcis* was 3.39 m/s in June, and *P. tangutica* was 2.447 m/s in March (Supplementary Table 8).

### Analysis of Fatty Acid Composition

**FIGURE 5:**
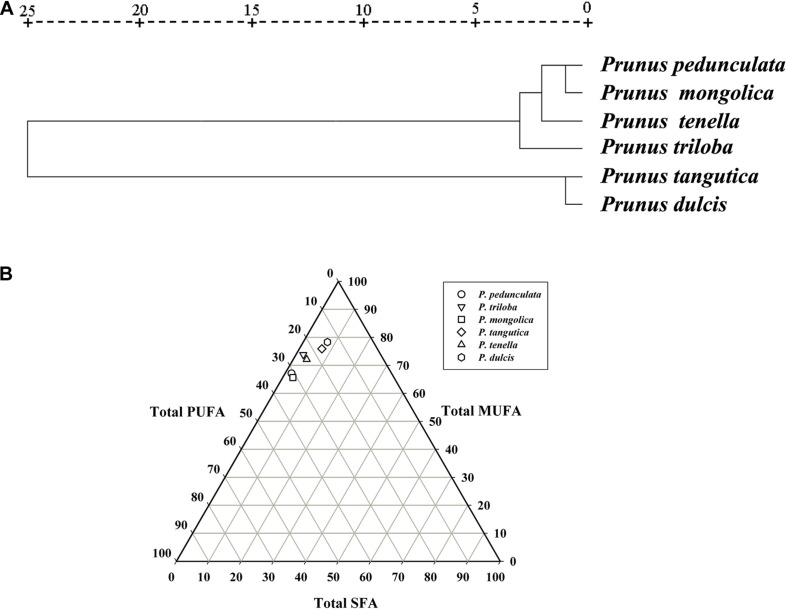
Cluster analysis of the fatty acids of the six almond species **(A)**; The trigonometric diagram of fatty acid composition (SFA, MUFA, and PUFA) **(B)**. Saturated fatty acid (SFA), monounsaturated fatty acid (MUFA), and polyunsaturated fatty acid (PUFA).

According to the clustering analysis of fatty acids, the six wild almond species can be divided into two groups (Figure 5A). Group-I consists of four species, *P. pedunculata*, *P. tenella*, *P. mongolica*, and *P. triloba*. Group-II consists of two species, *P. tangutica* and *P. dulcis*. Group- I has a low content of oleic acid (<75%) and a high content of linoleic acid (>20%). In contrast, Group-II has a high content of oleic acid (>75%) and a low content of linoleic acid (<20%) (Figure 5A and Supplementary Table 1).

The fatty acid composition including total saturated fatty acids (SFAs), monounsaturated fatty acids (MUFAs), and polyunsaturated fatty acids (PUFAs), is presented in Supplementary Table 1. When considering the content of polyunsaturated fatty acids, the six wild almond species could be divided into three groups: *P. tangutica* and *P. dulcis* below 20%, *P. tenella* and *P. triloba* between 20-30%, and *P. pedunculata* and *P. mongolica* above 30% (Figure 5B and Supplementary Table 1).

### Regions for the Potential Distribution of the Six Almond Species

The prediction of the six almond species potential distributions around the world consisted of three parts: (1) environmental variables, (2) model calibration and evaluation, and (3) tree species distribution.

#### MaxEnt Model Performance and Evaluation

Supplementary Figure 2 shows the omission rate and predicted area as functions of the cumulative threshold. The closer the observed omission rate is to the predicted omission rate, the more accurate the model simulation results are. Supplementary Figure 3 shows the receiver operating characteristic (ROC) curve for the same data. The evaluation metric results show that the test AUC values of all models were larger than 0.92, suggesting that the models worked well and had high prediction accuracy, but the values of change were not too large. The jackknife test evaluated the relative importance of environmental variables for each almond species and is presented in Supplementary Figure 4.

#### Analysis of the Bioclimatic Variables

Table 5 shows the evaluation of the relative contributions of the bioclimatic variables to the MaxEnt model for the six almond species selected for the analysis. For *P. triloba*, the contributions of Bio1, Bio15, and Bio3 were 37.8, 14.7, and 6.0%, respectively. The three bioclimatic variables with the greatest influence on the *P. tenella* distribution were Bio1 (33.9%), Bio15 (21.6%), and Bio10 (10.3%), accounting for 65.8% of the variation. The three most dominant variables for *P. tangutica* that had a cumulative contribution of 66.7% were screened as the key environmental factors: Bio12 (29.9%), Elev (24.3%), and Bio14 (12.5%). For *P. pedunculata*, the three most critical bioclimatic variables were Bio15 (33.9%), T_SAND (12.5%), and Bio10 (7.9%), accounting for 60.2% of the variation. The distribution of *P. mongolica* was significantly affected by Elev (15.1%), Bio18 (15%), and Bio13 (14.9%), accounting for 45% of the variation. For the *P. dulcis* distribution, the annual temperature annual range (Bio7, 23.6%) was second only to the mean temperature of the warmest quarter (Bio18, 26.9%), and the third was the mean temperature of the warmest quarter (Bio 10, 9.6%), accounting for 60.1% of the variation.

**TABLE 5:** Contribution of each environmental variable in MaxEnt modeling.

	*Prunus triloba*		*Prunus tenella*		*Prunus tangutica*		*Prunus pedunculata*		*Prunus mongolica*		*Prunus dulcis*	
Variable	Percent contribution	Permutation importance	Percent contribution	Permutation importance	Percent contribution	Permutation importance	Percent contribution	Permutation importance	Percent contribution	Permutation importance	Percent contribution	Permutation importance
Aspect	1.8	1.2	0.7	1.3	0.6	0.2	0.5	0	6.2	0.9	0.5	0.4
Bio1	37.8	24.3	33.9	32.3	0	0	3.9	0.6	0	0	3.6	1.4
Bio2	3.1	1.9	0.7	1.6	5.5	0.2	0.2	1.4	0.2	0.1	2	1.3
Bio3	6	2.4	2.1	3	7.3	1.6	0.4	7.7	0.4	0.6	0.2	1
Bio4	0.8	4.3	2.1	5.6	0.9	26.8	3.4	0	3.9	0.5	8.8	28.3
Bio5	2	0.8	0.2	1.1	0	0	0	0	1.4	0	1.1	0
Bio6	0.3	3.7	0.9	3.7	0	0	0.1	0.2	3.2	2.6	1.3	0.9
Bio7	2.8	0.2	8.9	7.6	1	0	1.7	0.4	3.3	0	23.6	0.4
Bio8	0.3	0.5	0.1	0.5	0	0	0	0	0	0	1.6	1
Bio9	1	13.1	1.1	2.9	0	0	2.9	13.7	0.2	1.9	6.3	3.8
Bio10	3.4	0.3	10.3	5.5	0	0.1	2.1	0.4	0.1	0.2	9.6	4
Bio11	1.2	4.5	1.8	1.2	0.3	0	0	3.7	3.5	11.7	2	2.3
Bio12	3.5	1.7	1	0.1	29.9	27.5	0	0	2	42.7	2.8	3.9
Bio13	0.2	0.7	0.8	9.2	0.2	4.9	5.7	14.6	14.9	17.8	0.3	0.1
Bio14	2	11.5	0.2	1.7	12.5	21.4	2.1	0	0.4	0.1	0.7	6.2
Bio15	14.7	4.4	21.6	4.7	5.2	3.7	39.8	49.2	0.6	1.2	0.6	2.5
Bio16	3.7	0.4	0.9	0.2	0	0	0	0	2.1	14.1	0.4	4.1
Bio17	2.8	2.5	0.3	1.8	0.1	0	2.7	1.4	0.1	0.2	0.7	3.3
Bio18	0.3	1.6	3.6	2.6	0	0	0.8	4	15	0	26.9	21.5
Bio19	1.9	1.5	0.2	2.7	0.5	13.2	3	0.3	7.6	1.8	0.5	4.1
Elev	2.7	15	0.7	1.5	24.3	0.1	7.9	0.7	15.1	0.5	1.1	4.7
Slope	2.7	0.9	2.8	6.7	1.4	0	0.5	0.5	3.1	2.3	1.3	2.7
T_OC	0.8	0.5	0.8	0.6	0.4	0	2.7	0.2	9.3	0.4	0.7	0.8
T_PH_H2O	0.6	0.6	1.6	0.9	9	0	4.8	0.5	0.1	0	0.4	0.3
T_SAND	2.2	0.4	2.2	0.7	0	0	12.5	0.6	0.3	0	2.9	0.8
T_SILT	1.5	1.3	0.4	0.6	0.9	0.2	2	0	7.1	0.3	0.4	0.4

However, the percentage of contribution values was only heuristically defined, depending on the particular path that the MaxEnt codes use to provide optimal solutions.

#### Prediction of the Potential Distribution of the Six Almond Species

**FIGURE 6:**
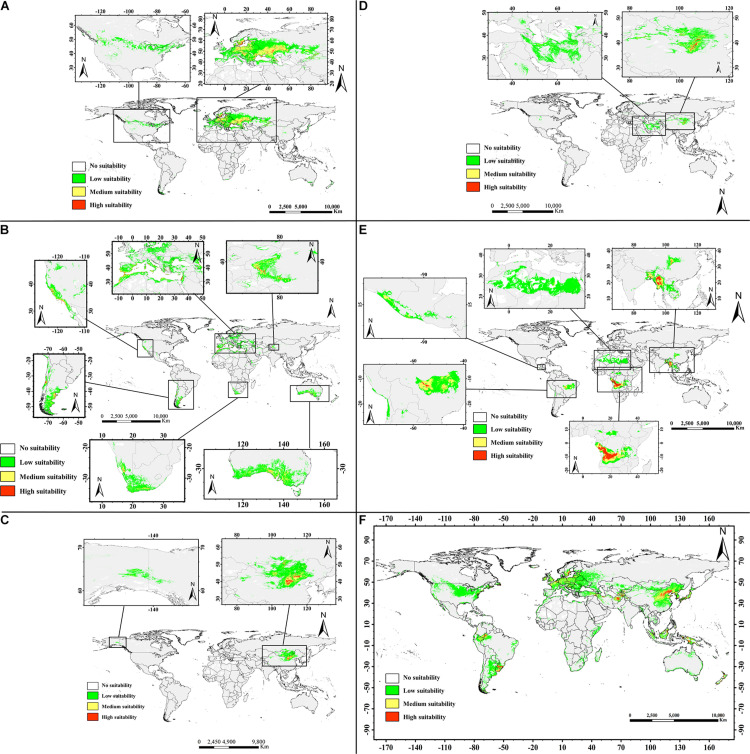
Predicted geographic distribution ranges for the almond species by MaxEnt in the world. Transparent to red color denotes the probability of occurrence from 0 to 1. Different habitat suitability ranks show different colors: transparent denotes no suitability, green means low suitability with probability of 0.05–0.33, yellow indicates medium suitability with probability of 0.33–0.66, and red represents high suitability with probability more than 0.66. (**A**, *P. tenella;* **B**, *P. dulcis;* **C**, *P*. *pedunculata;* **D**, *P. mongolica;* **E**, *P. tangutica;* and **F**, *P. triloba*).

Figures 6A–F shows the potential distributions of the six almond species, and the predicted output of the MaxEnt model values ranging from 0 to 1 was reclassified. The modeled results were reclassified into four levels using the Reclassify tool of ArcMap 10.2 for each almond species: (1). Unsuitable (0–0.05), (2). Generally, suitable (0.05–0.33), (3). Moderately suitable (0.33–0.66), and (4). Highly suitable (0.66–1). For each almond species under each defined class, the percentage area was calculated. According to the prediction results of the MaxEnt software, the moderately and highly suitable areas accounted for 8.08% of the total area of these six continents (Table 6).

**TABLE 6:** Percentage land surface area of different almond species habitat suitability types.

	Percentage of different areas (%)			
	Unsuitable habitat area	Low habitat suitability area	Moderate habitat suitability area	High habitat suitability area
*P. dulcis*	94.72	4.58	0.65	0.06
*P. mongolica*	98.30	1.56	0.13	0.02
*P. pedunculata*	98.10	1.54	0.30	0.07
*P. tangutica*	95.24	3.41	0.89	0.46
*P. tenella*	91.84	6.06	1.94	0.16
*P. triloba*	83.55	13.02	2.70	0.72
Total			8.08	

Suitable areas for *P. tenella* were found to be mainly distributed in western Asia and most of Europe, with smaller distributions in North America. There are very limited suitable distribution areas in the northwestern China (Figure 6A).

The suitable areas for *P. dulcis* were mainly distributed in southern Xinjiang in western China, the countries and regions around the Mediterranean, the west coast of the United States, southern South America, southern Africa and southern Australia (Figure 6B).

Regions suitable for *P. pedunculata* were concentrated in north-central China and parts of northwest China, the Middle East of Outer Mongolia, and parts of Russia bordering Outer Mongolia on the world map. There are very few suitable areas in northwest North America. The overall suitable range is relatively narrow (Figure 6C).

The suitable areas for *P. mongolica* were mainly distributed in the north-central part of China and the south-central part of Outer Mongolia, as well as some areas in western Asia with low suitability. The suitable range is narrow (Figure 6D).

The prediction results of the MaxEnt model indicated that the suitable area of *P. tangutica* around the world was very wide, and the suitable areas were mainly concentrated in western Sichuan Province in China, south Asia, West-Central Africa and the Middle East of South America; in addition, there were very wide low suitability areas in northern Africa. In general, the suitable distribution area of *P. tangutica* was also wide (Figure 6E).

According to the prediction for *P. triloba*, there are many suitable distribution areas in eastern China, western and southern Asia, Europe, southeastern Asia, and northwestern South America. In addition, there are some low suitability distribution areas in northern United States and around Australia. On the whole, its suitable distribution area is relatively wide (Figure 6F).

The percentage areas of different habitat suitability types of each almond species are shown in Table 6. The high habitat suitability area of the almond species accounts for 0.02–0.72% of the global ecologically suitable distribution area, with *P. triloba* as the highest and *P. mongolica* as the lowest; the moderate habitat suitability area is 0.13–2.70%, among which *P. triloba* is the highest and *P. mongolica* is the lowest; the low habitat suitability area is *P. triloba* with the highest (13.02%) and *P. pedunculata* the lowest (1.54%).

### Phylogenetic Analysis Based on the Chloroplast Genome of the Six Almond Species

**FIGURE 7:**
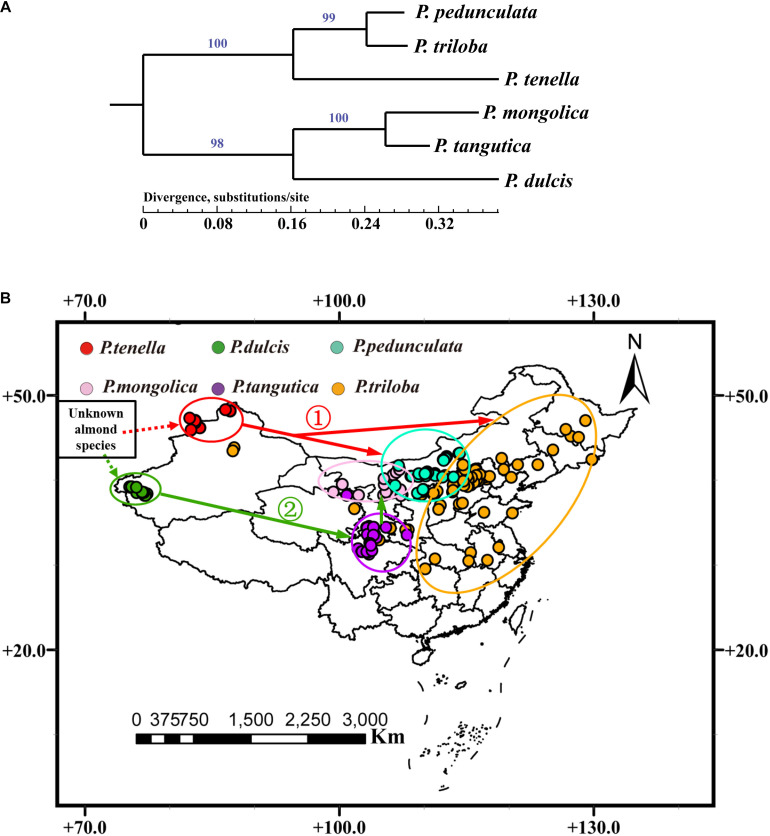
**(A)** Phylogenetic relationships of six almond species inferred from maximum likelihood (ML) analysis based on whole chloroplast genome sequences. Numbers above nodes are support values with Maximum Likelihood (ML) analyses; **(B)** A possible model for the evolution of the six almond species in China.

In the present study, six complete chloroplast genomes of almond species (Supplementary Table 9) were used to construct the phylogenetic trees. It was clear that the six species were classified into two groups. As Figure 7A illustrates, one group, *P. triloba* was closer to *P. pedunculata* than to *P. tenella*; the other group, *P. tangutica* was closer to *P. mongolica* than to *P. dulcis*.

Further combined with the distribution of almond species in China, the six almond species may have evolved from an “unknown almond species” through two routes (Figure 7B). The first route evolved from an “unknown almond species” to *P. tenella*, and then to *P. pedunculata* and *P. triloba* (Figure 7B ➀); the other route evolved from an “unknown almond species” to *P. dulcis*, and then to *P. tangutica* and *P. mongolica* (Figure 7B ➁).

## Discussion

### Importance of the Current Situation, Global Potential Distribution and Evolution

Almonds (*P. dulcis*) are among the most popular tree nuts around the world and can be beneficial for human health and nutrition (Čolić et al., 2017). Wild almond genetic resources have also received considerable attention for their nut nutritional value and medicinal use (Esfahlan and Jamei, 2012; Mirzapour et al., 2017; Wang et al., 2018b, 2019). Here, we investigated six almond species in China to understand the distribution characteristics of almond resources and to provide the basis for further protection and development. This is the first study to model almond species in China’s potential distributions in combination with fatty acid composition analysis and phylogenetic analysis. These results could provide a basis for the cultivation and utilization of important economic plants.

### Ecological Characteristics of the Almond Species

Different plants need different climates and habitats for their growth. In this study, the environment of the *P. dulcis* almond habitat was investigated, and it was found that the annual mean temperature was 15.08°C. The annual precipitation of *P. dulcis* is mainly in the winter. The suitable distribution areas for *P. dulcis* have a typical Mediterranean climate providing a dry climate in summer and milder winter rain climate conditions. *P. dulcis* also shows high tolerance to summer drought and heat. Almond yield was positively affected by annual mean temperature and annual precipitation (Ighbareyeh et al., 2018). Therefore, understanding the climate data of almond is of great practical significance for almond cultivation.

*Prunus tangutica* is a unique almond species in China that is mainly distributed in the northwestern Sichuan Province, with an altitude of 939–4878 m, an annual average temperature of 7.2°C, 671.9 mm annual precipitation and water vapor pressure ranging from 0.29 to 1.41 kPa. *P. tangutica* prefers cold and semihumid climate conditions with low latitudes and high altitudes.

The current distribution areas for *P. mongolica* have a typical cold temperate continental monsoon climate, providing cold, drought, and strong light climate conditions. The lowest annual precipitation is 173.9 mm in the natural distribution area of *P. mongolica*, which may be the most drought-resistant of the six species.

*Prunus pedunculata* has great ornamental and medicinal value (Wang et al., 2018b) and is located in northwest China, with an annual average temperature of –5.18 to 7.82°C, and an annual precipitation of 52.0–416.0 mm. The geographical distribution areas of *P. pedunculata* and *P. mongolica* are relatively close, and their climatic conditions are similar, but the annual precipitation in the *P. mongolica* distribution is lower, and the annual average temperature in the *P. pedunculata* distribution is lower.

The main area of *P. tenella* is located in the southwestern of Russia, Kazakhstan, Sweden, Ukraine, and northwestern China, with latitudes of 41.86 to 60.47°N, an annual average temperature of 5.53°C. *P. tenella* prefers cold and dry climate conditions at high latitudes.

The genus *P. triloba* is distributed all over the world, ranging from the eastern parts of China to Central America. Among these six almond species, *P. triloba* is mostly found at longitudes ranging from 117.33°W to 129.79°E, within 29.54°N –59.87°N latitudes, 5.00–2438.00 m altitudinal regions and 186.0–1541.0 mm annual precipitation. Based on the current distribution, *P. triloba* is the most widely dispersed species among the six species, which also shows its high environmental adaptability.

### Relationship Between Fatty Acid Composition and Geographical Distribution

Previous studies have shown that environmental factors such as elevation, maximum temperature, and precipitation have significant effects on the variation in plant seed kernel oil fatty acid composition among populations (Akashi et al., 2017; Sun et al., 2017). Our previous study found that the fatty acids of five wild almond resources were different (Wang et al., 2019). In this study, combined with cultivated almond, the fatty acid cluster analysis results of six almond resources are basically consistent with their geographical distribution: the fatty acid composition of *P. dulcis* and *P. tangutica* in the low latitude distribution is similar, while the other four almond species in the cold temperate region have similar fatty acids. These results showed that the composition of fatty acids was not only related to varieties but also closely related to the growth environment. This provides a reference for the purposeful production of fatty acids with different characteristics in different regions.

### Potential Distribution of the Six Almond Species Around the World

The ROC curve is not affected by the threshold and is considered to be one of the best evaluation indicators at present. The ROC area under the curve (AUC) method is a widely used procedure for comparing the species distribution model performances of prediction models (Busby, 1991; Carpenter et al., 1993; Stockwell and Peters, 1999; Phillips et al., 2006). MaxEnt software can directly draw the ROC curve and calculate the AUC value of the model, which is convenient for judging the predictive effect of the model. Therefore, ROC curves are widely used in the evaluation of MaxEnt models. Li et al. (2020) used ROC curves to evaluate the predictive effect of the MaxEnt model in terms of suitable habitats for three Coptis herbs in China (Li et al., 2020), and Gilani et al. (2020) used ROC curves to determine the accuracy of niche models in predicting suitable habitats for six native tree species in Gilgit-Baltistan, Pakistan (Gilani et al., 2020).

The AUC, a value between 0 and 1, represents the probable accuracy of the model simulation. The model is considered useful when the AUC value is greater than 0.75, and the predicted result will be excellent if the AUC value is between 0.9 and 1 (Phillips et al., 2006). In the present study, the MaxEnt model for each almond species provided satisfactory results, with an AUC training and testing values greater than 0.92. This indicates that the model can be used to simulate the potential distribution of the six almond species. The jackknife test of variables influencing the overall temperature, precipitation, and elevation are examples of factors with the highest gain. The contribution of the least variables varied for each almond species.

The MaxEnt model has predicted that different species have different potentially distributed suitable areas (Zhang et al., 2018, 2019; Gilani et al., 2020; Li et al., 2020). The model results showed that under the current climatic conditions, the environmental suitability of *P. dulcis* lies within southern Xinjiang in China, the countries around the Mediterranean, the west coast of the United States, southern South America, southern Africa and southern Australia. This finding fits with our field observations and the known distribution reported in the literature (Browicz and Zohary, 1996; Ladizinsky, 1999).

In 3,000 BC, domesticated almond (sweet-seeded) was in use in Mediterranean civilizations. Since that time, the almond kernel has been an edible part of the nut and is considered to be an important food crop with high nutritional value (Company et al., 2010). In the past 30 years, *P. dulcis* has been widely cultivated. The suitable distribution area of almond in this study can provide an important reference for its introduction.

Based on the results, *P. tenella*, *P. mongolica*, and *P. pedunculata* have broad application prospects in desertification control. They can be cultivated in most cold and dry areas as oil crops (Wang et al., 2019), which also helps farmers to generate more income. Finally, a plantation program in suitable areas will enhance the area to be more productive and beneficial for farmers and the environment.

The most suitable regions for *P. tangutica* are in the low latitude region (30°N to 20°S). Among the six almond species, *P. tangutica* is the most suitable for low latitude and high altitude areas. Our results suggest that a larger area is more climatically suitable for *P. triloba* introduction and cultivation worldwide than its current distribution.

### Evolutionary Relationship of Almond Species Based on the Phylogenetic Analyses of the Chloroplast Genome

The chloroplast organelle is the site of photosynthesis and carbon fixation in plants. Chloroplast DNA (cpDNA) has become an effective tool for the study of plant genetic evolution and the identification of interspecific and intraspecific polymorphisms because of its unique maternal inheritance and low silent nucleotide substitution rate (Zeng et al., 2017; Wang et al., 2018a). Our previous research results showed that the phylogenetic tree was constructed with the complete chloroplast genomes, and the genetic relationship of amygdala was clarified (Wang et al., 2018a, 2020). Based on the analysis of chloroplast DNA (cpDNA) evolution, the six almond species can be divided into two parallel groups, which is consistent with their latitudinal geographical distribution. *P. pedunculata*, *P. triloba*, and *P. tenella* are distributed at relatively high latitudes, and *P. mongolica*, *P. tangutica*, and *P. dulcis* are distributed at relatively low latitudes. There were no initial species in the two groups, so it is inferred that the six almond species distributed in China may have originated from an “unknown almond species” in western Asia. This is consistent with previous reports that western countries such as Iran (Esfahlan and Jamei, 2012; Sorkheh et al., 2016) are within the center of origin of almond. However, the origin of almond resources in China needs further study. In addition, combined with the results of almond species surveys and prediction distribution analysis, China may not be the center of origin of almond resources, but almond species, especially wild almond species, are widely distributed in China.

Among the six almond species, *P. mongolica* is the narrowest in its current distribution area and in its predicted distribution area. *P. mongolica* is closely related to *P. dulcis* which is not resistant to low temperatures and *P. tangutica* which is distributed at low latitudes, so it is speculated that its cold resistance is not as good as that of *P. pedunculata* and *P. tenella*, which may be the reason why *P. mongolica* is not suitable for distribution in the cold temperate zone at high latitudes. This species has been recorded as a rare plant on the China Plant Red List and adopted as a state key conservation species (Yazbek and Al-Zein, 2014).

## Conclusion

Based on first-hand survey data, combined with global resource distribution data, the geographical distribution of the six almond species in China was investigated and analyzed. The results indicated that different plants need different climates and habitats for their growth. The suitable distribution areas for *P. dulcis* have a typical Mediterranean climate. *P. tangutica* prefers cold and semihumid climate conditions with low latitudes and high altitudes. *P. mongolica* has a typical cold temperate continental monsoon climate preference that provides cold, drought, and strong light conditions. The geographical distribution areas of *P. pedunculata* and *P. mongolica* are relatively close and their climatic preferences are similar. *P. tenella* prefers cold and dry climate conditions at high latitudes. *P. triloba* is the most widely dispersed species among the six species. The climate of the almond species distribution areas have specific characteristics, and the distribution of almond species is consistent with the results of fatty acid cluster analysis.

The MaxEnt model for each almond species provided satisfactory results. The prediction results can provide an important reference for *P. dulcis* cultivation, wild almond species development and protection. A plantation program in a suitable area will enhance the area to be more productive and beneficial for the farmers and the environment. Based on these results, *P. tenella*, *P. mongolica*, and *P. pedunculata* have broad application prospects in desertification control. Regarding these results, most of the cold and dry areas can cultivate almond as oil crops, which also helps farmers to generate more income.

In this study, a phylogenetic tree based on the chloroplast genome and the characteristics of geographical distribution was constructed. The six almond species in China may have evolved from an “unknown almond species” through two routes. Although almond resources are widely distributed in China, the Chinese mainland may not be the origin of all almond species. The prediction of the evolution of these six almond species will expand the researchers’ vision of almond species diversity and promote an understanding of the evolutionary relationships among the various almond species.

## Data Availability Statement

The original contributions presented in the study are included in the article/Supplementary Material, further inquiries can be directed to the corresponding author/s.

## Author Contributions

WW conceived and X-QX designed the study. WW processed the data, performed the analyses and analyzed the results, and wrote the manuscript. Z-JL, Y-LZ, and X-QX edited the manuscript. All authors read and approved the final version of the manuscript.

## Conflict of Interest

The authors declare that the research was conducted in the absence of any commercial or financial relationships that could be construed as a potential conflict of interest.

## Footnotes

1 https://www.gbif.org

2 www.worldclim.org

3 http://www.fao.org/soils-portal/soil-survey/soil-maps-and-databases/harmonized-world-soil-database-v12/en/

4 http://www.atgc-montpellier.fr/phyml/
