# Supplementary figures and images for: Current Situation, Global Potential Distribution and Evolution of Six Almond Species in China
Source: Front Plant Sci. 2021 Apr 23;12:619883. doi: 10.3389/fpls.2021.619883 (PMC8102835; doi:10.3389/fpls.2021.619883)

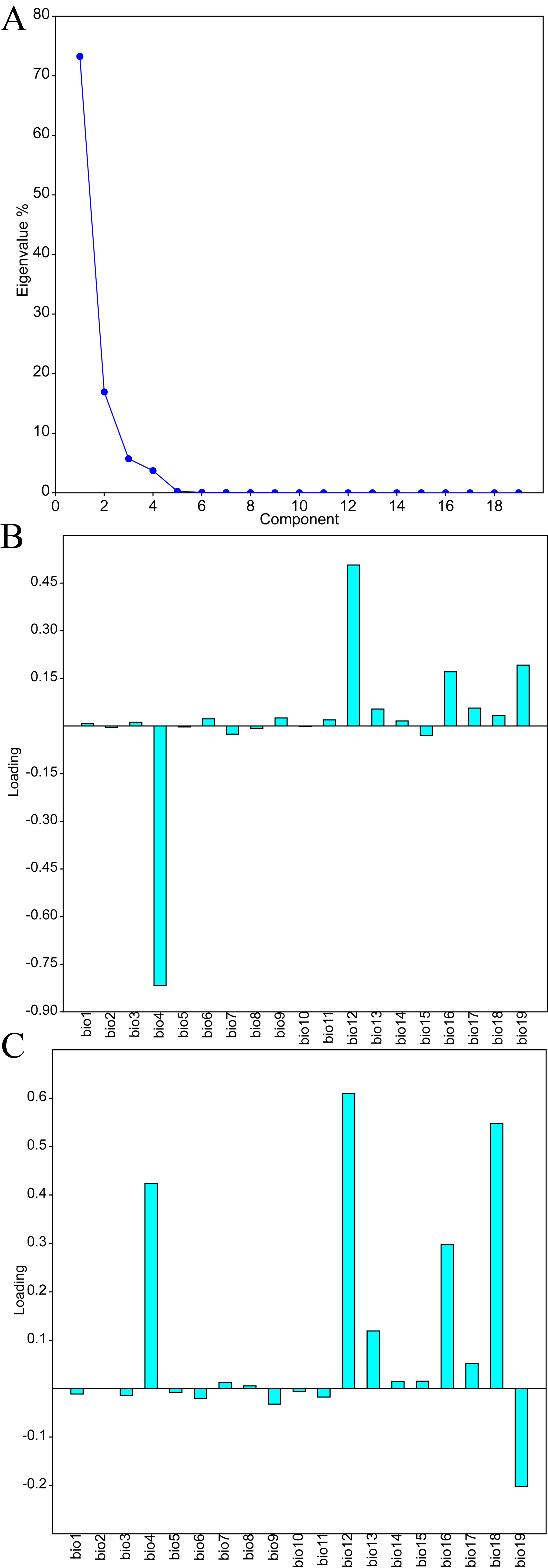

Supplement: Supplementary file 1 [file Image_1.JPEG]

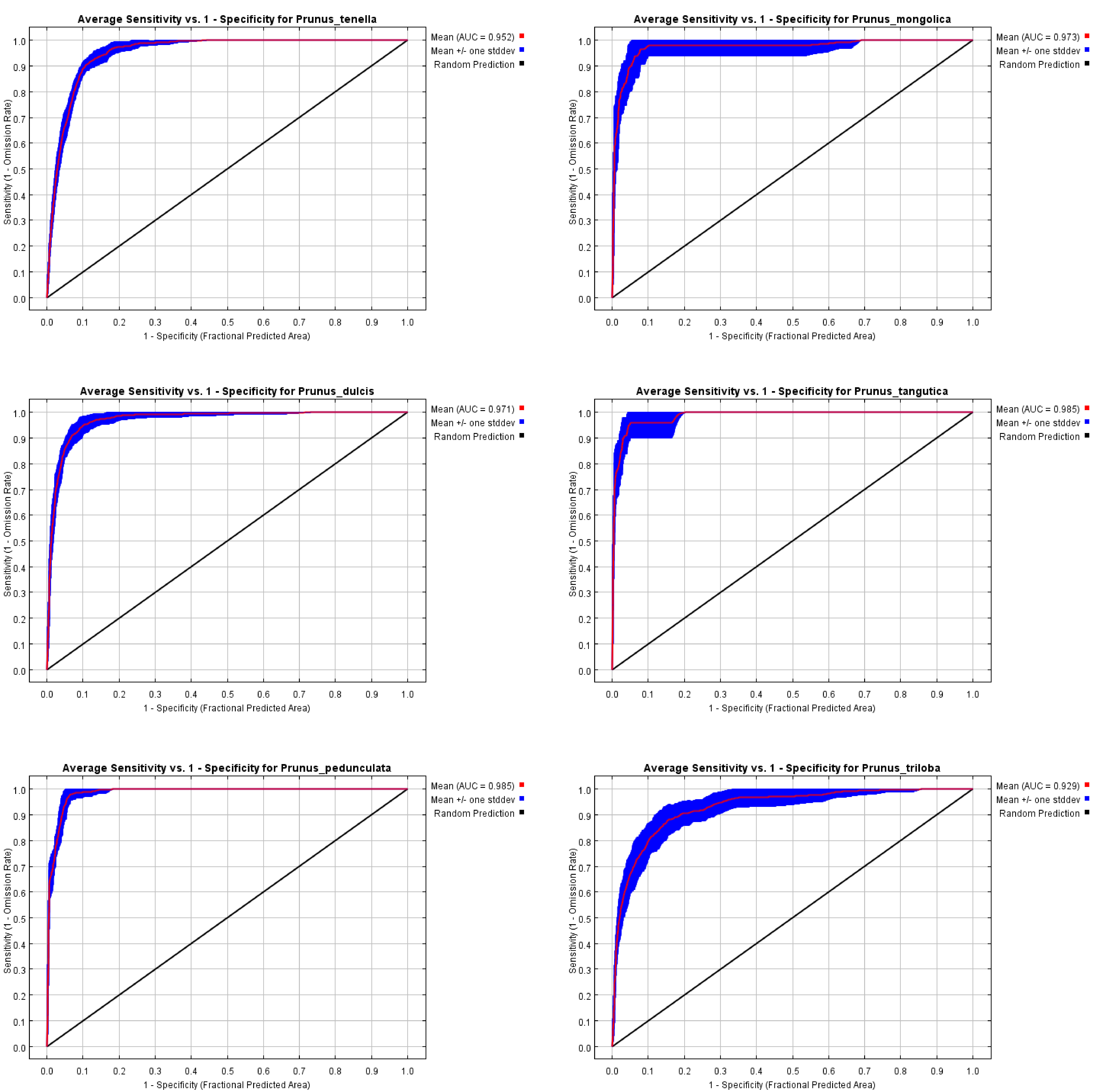

Supplement: Supplementary file 2 [file Image_2.JPEG]

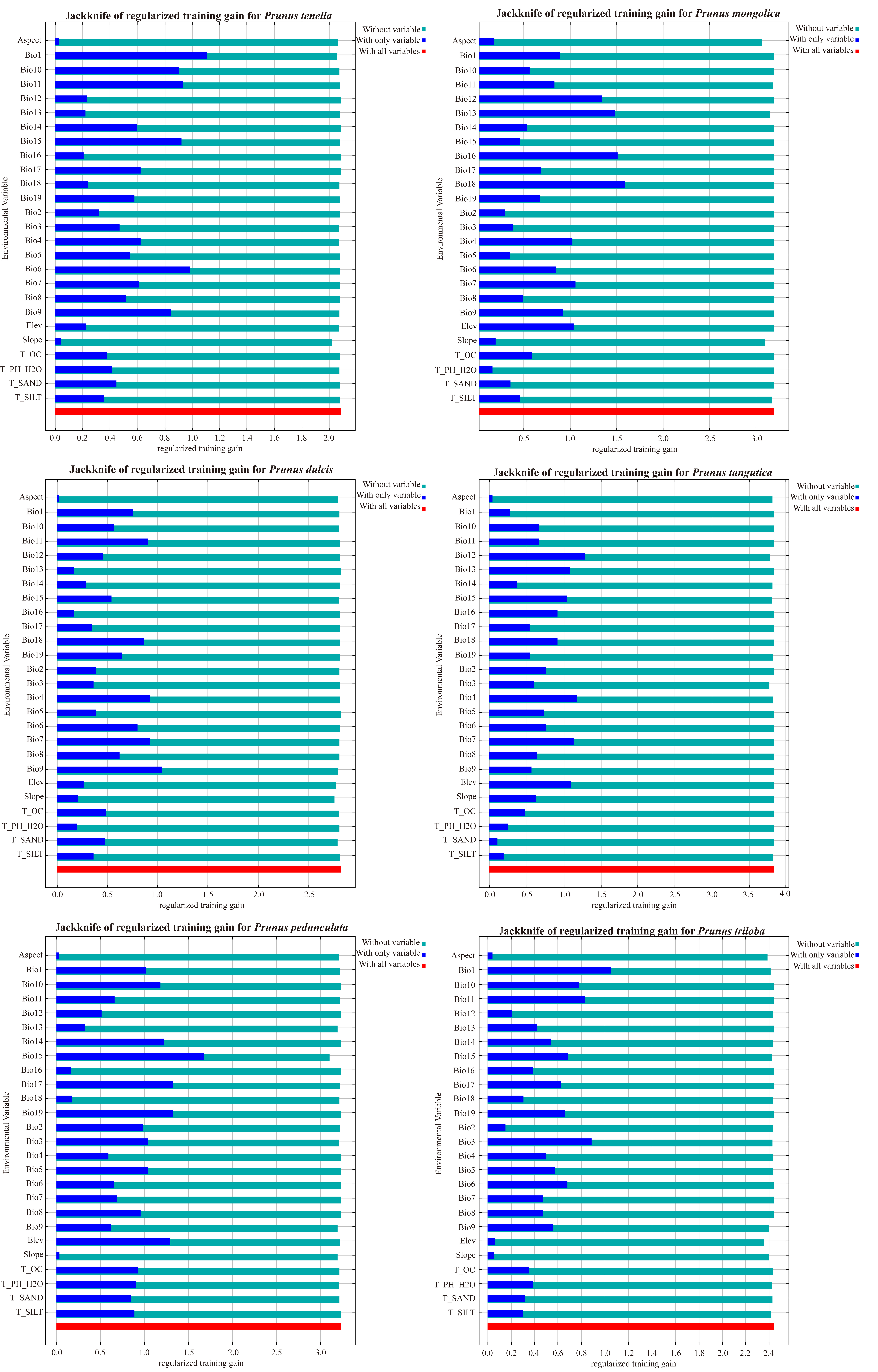

Supplement: Supplementary file 3 [file Image_3.JPEG]

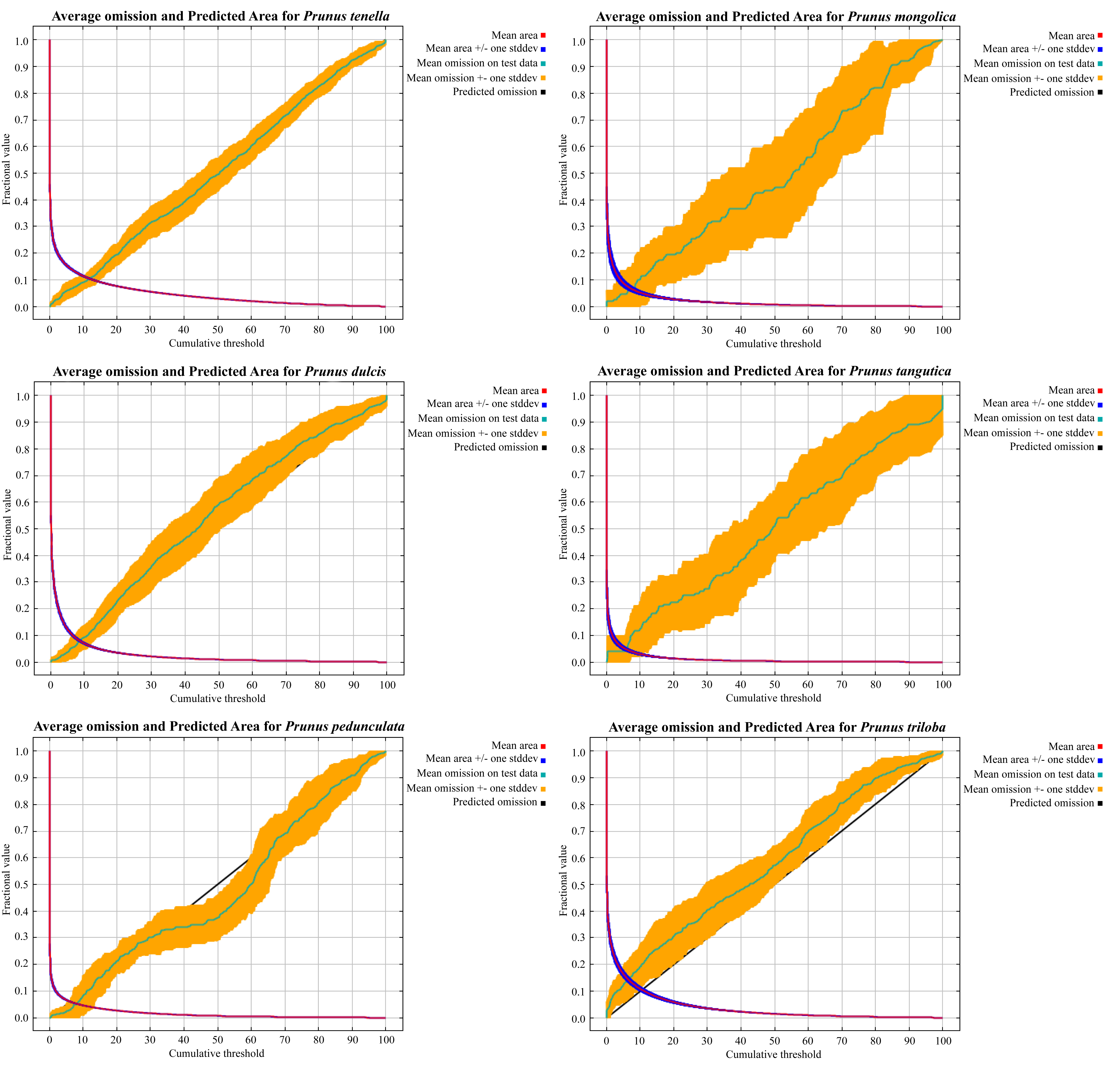

Supplement: Supplementary file 4 [file Image_4.JPEG]
