# Supplementary material for: Current Situation, Global Potential Distribution and Evolution of Six Almond Species in China
Source: Front Plant Sci. 2021 Apr 23;12:619883. doi: 10.3389/fpls.2021.619883 (PMC8102835; doi:10.3389/fpls.2021.619883)
Supplement: Supplementary file 5 [file Data_Sheet_1.docx]

Table S1 Oil content in six almond species and fatty acids composition of oil.

|  | *P. pedunculata* | *P. triloba* | *P. mongolica* | *P. tangutica* | *P. tenella* | *P. dulcis* |
| --- | --- | --- | --- | --- | --- | --- |
| Oil contents % | 50.4 | 46.3 | 49.5 | 43.6 | 45.9 | 54.8 |
| Fatty acids composition (%) |  |  |  |  |  |  |
| Saturated fatty acids |  |  |  |  |  |  |
| Palmitic C16:0 | 1.5 | 1.8 | 2.4 | 5 | 3.1 | 6.2 |
| Margaric C17:0 | — | — | — | 0.1 | — | 0.1 |
| Stearic C18:0 | 0.5 | 0.5 | 0.8 | 1.9 | 1.1 | 1.2 |
| Arachidic C20:0 | — | — | — | 0.1 | — | — |
| Monounsaturated fatty acid |  |  |  |  |  |  |
| Palmitoleic C16:1 | 0.2 | 0.2 | 0.2 | 0.2 | 0.3 | 0.5 |
| Heptadecenoic C17:1 | 0.1 | 0.1 | 0.1 | 0.1 | 0.1 | 0.1 |
| Oleic (18:1) | 66.5 | 73.1 | 65.3 | 75.4 | 71.7 | 77.8 |
| cis-11-Eicosenoic (20:1) | 0.2 | 0.1 | 0.1 | 0.1 | 0.1 | 0.1 |
| Polyunsaturated fatty acid |  |  |  |  |  |  |
| Linoleic C18:2 | 30.8 | 23.8 | 31.1 | 17 | 23.6 | 14.2 |
| α-Linolenic C18:3 | 0.1 | 0.1 | 0.1 | 0.1 | 0.1 | — |
| Others | 0.1 | 0.1 | — | 0.1 | — | — |
| Saturated fatty acids | 2 | 2.3 | 3.2 | 7 | 4.2 | 7.5 |
| Monounsaturated fatty acid | 67 | 73.5 | 65.7 | 75.8 | 72.2 | 78.5 |
| Polyunsaturated fatty acid | 30.9 | 23.9 | 31.2 | 17.1 | 23.7 | 14.2 |
| Total unsaturated fatty acids | 97.9 | 97.4 | 96.9 | 93 | 95.9 | 92.7 |
| Source | S. Afr. J. Bot. 121, 274-281 (2019) | S. Afr. J. Bot. 121, 274-281 (2019) | S. Afr. J. Bot. 121, 274-281 (2019) | S. Afr. J. Bot. 121, 274-281 (2019) | S. Afr. J. Bot. 121, 274-281 (2019) | Unpublished |

Table S2 Bioclimatic profiles of the six almond species based on the each month of the year (January is 1; December is 12) for average temperature (°C).

|  | *P. pedunculata* | *P. triloba* | *P. mongolica* | *P. tangutica* | *P. tenella* | *P. dulcis* |
| --- | --- | --- | --- | --- | --- | --- |
| 1 | -25.0--9.4(-12.518±0.1444) | -21.0-13.3(-5.737±0.5242) | -16.6--7.7(-9.697±0.1709) | -15.3-1.0(-3.442±0.1896) | -18.5-5.2(-8.685±0.2629) | -9.5-13.6(6.356±0.3511) |
| 2 | -20.4--5.2(-8.351±0.1517) | -18.4-14.2(-3.610±0.4651) | -13.6--3.7(-6.195±0.1873) | -12.9-3.4(-0.823±0.1956) | -15.8-5.8(-7.879±0.2489) | -7.0-13.7(7.579±0.3271) |
| 3 | -13.7-1.8(-0.930±0.1304) | -9.4-14.9(1.914±0.3563) | -8.2-3.0(0.645±0.1995) | -8.8-7.6(3.464±0.2083) | -11.2-7.9(-2.902±0.2078) | -2.2-14.8(10.298±0.2636) |
| 4 | -5.2-9.8(7.462±0.1141) | -0.8-17.5(8.801±0.2869) | -2.0-10.9(8.765±0.1961) | -4.9-12.4(8.242±0.2243) | 1.1-12.3(6.446±0.1068) | 3.8-18.6(13.825±0.2398) |
| 5 | 2.1-16.5(14.421±0.0932) | 7.1-22.3(14.705±0.2548) | 3.1-17.9(15.491±0.2091) | -0.9-16.0(11.669±0.2216) | 7.6-17.7(12.928±0.0923) | 9.1-22.4(17.777±0.2163) |
| 6 | 7.6-21.4(19.113±0.0835) | 10.8-26.0(18.905±0.2644) | 6.9-22.9(20.224±0.2259) | 2.3-19.6(14.348±0.2229) | 12.3-24.3(17.409±0.0928) | 13.4-25.7(21.255±0.1996) |
| 7 | 9.7-23.9(21.412±0.0823) | 13.9-29.3(21.071±0.2665) | 9.3-24.9(22.404±0.2288) | 4.6-21.7(16.341±0.2189) | 14.9-25.8(19.594±0.0956) | 16.3-27.7(23.491±0.1920) |
| 8 | 8.2-21.6(19.405±0.0897) | 13.7-28.8(20.021±0.2652) | 8.4-23.3(20.729±0.2138) | 4.4-21.3(16.051±0.2086) | 13.5-24.4(18.263±0.0981) | 15.3-27.6(23.389±0.2027) |
| 9 | 2.6-15.9(13.714±0.0930) | 8.3-24.1(15.263±0.2747) | 4.1-17.6(15.175±0.1981) | 1.6-16.4(12.160±0.1909) | 8.0-19.6(13.002±0.0935) | 10.6-26.1(20.830±0.2469) |
| 10 | -4.8-8.4(6.330±0.1037) | -0.4-20.5(9.274±0.2896) | -2.5-9.5(7.675±0.1792) | -3.0-11.9(7.790±0.1940) | .2-14.7(6.005±0.1207) | 6.0-22.9(16.771±0.2895) |
| 11 | -12.6--0.1(-2.869±0.1278) | -11.0-16.4(1.797±0.3925) | -10.0-1.4(-1.003±0.1838) | -8.4-7.1(2.597±0.1990) | -9.6-10.1(-1.509±0.1996) | -1.7-17.7(11.629±0.3102) |
| 12 | -20.7--7.2(-10.381±0.1430) | -17.3-13.5(-3.770±0.4962) | -14.8--5.3(-7.603±0.1691) | -13.3-2.6(-2.037±0.1851) | -15.0-6.8(-6.303±0.2460) | -7.6-14.8(7.713±0.3336) |

Table S3 Bioclimatic profiles of the six almond species based on the each month of the year (January is 1; December is 12) for maximum temperature (°C).

|  | *P. pedunculata* | *P. triloba* | *P. mongolica* | *P. tangutica* | *P. tenella* | *P. dulcis* |
| --- | --- | --- | --- | --- | --- | --- |
| 1 | -19.40--1.80(-5.52±0.17) | -15.40-20.00(-0.64±0.45) | -7.90--0.50(-3.32±0.22) | -8.10-7.80(2.47±0.16) | -19.40-20.00(-3.63±0.14) | -4.10-17.10(10.62±0.36) |
| 2 | -13.30-2.10(-1.22±0.16) | -13.60-20.80(1.80±0.41) | -4.90-3.40(0.50±0.23) | -5.70-10.50(4.90±0.17) | -13.60-20.80(-0.93±0.13) | -1.10-18.70(12.14±0.34) |
| 3 | -8.40-8.70(5.91±0.13) | -3.70-21.00(7.41±0.34) | 0.00-10.40(7.34±0.24) | -2.70-14.10(9.06±0.19) | -8.40-21.00(4.97±0.12) | 2.80-20.70(15.25±0.29) |
| 4 | 0.00-17.50(14.92±0.13) | 4.40-24.40(14.86±0.32) | 5.40-18.90(15.90±0.24) | 0.30-18.10(14.04±0.22) | 0.00-24.40(13.72±0.09) | 8.20-25.30(19.46±0.27) |
| 5 | 7.40-24.20(21.86±0.10) | 12.00-27.40(20.96±0.27) | 9.80-25.70(22.57±0.25) | 4.10-21.20(17.40±0.23) | 4.10-27.40(20.22±0.08) | 14.60-30.20(23.81±0.25) |
| 6 | 12.90-28.80(26.17±0.09) | 17.60-32.20(24.79±0.28) | 13.10-30.50(27.03±0.26) | 6.90-24.20(19.63±0.23) | 6.90-32.20(24.29±0.09) | 18.60-34.00(27.40±0.23) |
| 7 | 14.50-30.60(27.75±0.09) | 20.80-34.30(26.42±0.26) | 15.40-32.10(28.79±0.25) | 9.50-25.90(21.64±0.22) | 9.50-34.30(26.15±0.09) | 20.40-35.90(29.70±0.22) |
| 8 | 13.10-28.00(25.42±0.10) | 19.70-34.50(25.42±0.26) | 15.00-30.10(26.82±0.24) | 9.70-25.70(21.59±0.20) | 9.70-34.50(24.69±0.08) | 19.80-34.90(29.49±0.23) |
| 9 | 7.40-22.90(20.33±0.10) | 12.80-32.10(21.00±0.29) | 10.60-24.80(21.63±0.23) | 6.40-20.60(16.87±0.19) | 6.40-32.10(19.49±0.08) | 14.70-31.60(26.81±0.28) |
| 10 | -0.30-15.40(13.15±0.12) | 2.90-27.90(14.87±0.31) | 4.60-17.20(14.30±0.22) | 1.90-16.30(12.40±0.18) | -0.30-27.90(12.44±0.08) | 9.20-28.50(22.35±0.33) |
| 11 | -7.80-6.90(3.71±0.15) | -7.10-23.80(6.75±0.37) | -1.90-8.20(5.16±0.23) | -2.10-12.10(8.01±0.18) | -7.80-23.80(4.06±0.11) | 3.80-23.20(16.56±0.33) |
| 12 | -15.70-0.00(-3.75±0.17) | -12.40-20.60(1.10±0.44) | -6.10-1.10(-1.63±0.21) | -6.30-7.70(3.88±0.16) | -15.70-20.60(-1.82±0.13) | -1.60-18.00(12.04±0.34) |

Table S4 Bioclimatic profiles of the six almond species based on the each month of the year (January is 1; December is 12) for minimum temperature (°C).

|  | *P. pedunculata* | *P. triloba* | *P. mongolica* | *P. tangutica* | *P. tenella* | *P. dulcis* |
| --- | --- | --- | --- | --- | --- | --- |
| 1 | -30.70--16.40(-19.51±0.12) | -27.30-6.70(-10.84±0.62) | -25.30--14.00(-16.07±0.15) | -22.60--3.80(-9.39±0.23) | -23.50-2.40(-12.44±0.30) | -15.00-11.00(2.10±0.36) |
| 2 | -28.00--12.50(-15.49±0.14) | -23.30-7.70(-9.02±0.54) | -22.30--10.30(-12.92±0.16) | -20.10--1.50(-6.57±0.23) | -21.20-2.40(-12.00±0.28) | -13.30-10.70(3.02±0.33) |
| 3 | -18.90--5.10(-7.76±0.13) | -15.20-8.80(-3.58±0.40) | -16.40--3.60(-6.05±0.17) | -14.90-2.60(-2.14±0.23) | -18.90-4.20(-7.28±0.23) | -7.20-11.10(5.34±0.25) |
| 4 | -10.30-2.10(0.01±0.10) | -5.90-13.10(2.74±0.29) | -9.30-3.50(1.62±0.17) | -10.20-7.20(2.45±0.23) | -4.70-8.60(1.18±0.11) | -0.70-12.80(8.20±0.22) |
| 5 | -3.20-8.80(7.00±0.09) | 1.90-17.80(8.46±0.26) | -3.60-10.00(8.39±0.18) | -5.90-11.10(5.95±0.22) | 1.00-12.40(6.91±0.10) | 3.70-16.40(11.76±0.20) |
| 6 | 2.20-14.40(12.05±0.08) | 3.90-21.90(13.02±0.27) | 0.70-15.40(13.41±0.20) | -2.30-14.90(9.07±0.22) | 5.50-17.40(11.39±0.09) | 7.30-20.80(15.10±0.19) |
| 7 | 4.90-18.20(15.10±0.08) | 5.70-25.20(15.71±0.29) | 3.10-18.30(16.03±0.22) | -0.30-17.50(11.03±0.22) | 8.10-19.40(13.60±0.09) | 10.60-23.10(17.28±0.19) |
| 8 | 3.40-15.70(13.38±0.08) | 4.90-24.60(14.62±0.29) | 1.90-16.50(14.62±0.20) | -1.00-17.00(10.56±0.22) | 6.10-19.10(12.14±0.10) | 10.00-23.20(17.27±0.21) |
| 9 | -2.20-8.80(7.10±0.09) | 2.00-19.70(9.53±0.29) | -2.50-10.40(8.72±0.17) | -3.10-12.60(7.48±0.20) | 0.90-15.00(7.30±0.10) | 5.40-20.70(14.85±0.24) |
| 10 | -9.30-1.40(-0.49±0.09) | -3.70-14.20(3.66±0.32) | -9.60-2.70(1.07±0.15) | -8.00-7.70(3.19±0.20) | -5.80-10.40(1.34±0.14) | -0.50-17.30(11.19±0.28) |
| 11 | -20.00--7.10(-9.44±0.11) | -14.90-9.00(-3.14±0.45) | -18.10--4.00(-7.14±0.16) | -15.00-2.60(-2.79±0.22) | -13.70-6.60(-5.01±0.23) | -8.00-14.10(6.70±0.31) |
| 12 | -26.10--13.70(-17.04±0.12) | -23.10-6.40(-8.65±0.58) | -23.40--10.70(-13.58±0.15) | -20.30--1.90(-7.97±0.22) | -19.70-3.80(-9.72±0.28) | -13.60-12.10(3.39±0.34) |

Table S5 Bioclimatic profiles of the six almond species based on the each month of the year (January is 1; December is 12) for precipitation (mm).

|  | *P. pedunculata* | *P. triloba* | *P. mongolica* | *P. tangutica* | *P. tenella* | *P. dulcis* |
| --- | --- | --- | --- | --- | --- | --- |
| 1 | 1.0-10.0(3.427±.0557) | 0.0-124.0(23.771±2.2968) | 0.0-4.0(1.771±.1020) | 1.0-9.0(3.139±.0959) | 6.0-155.0(29.272±.8131) | 0.0-208.0(83.079±3.0638) |
| 2 | 0.0-6.0(3.789±.0313) | 2.0-102.0(22.301±1.9750) | 0.0-4.0(1.677±.0804) | 2.0-12.0(7.036±.1652) | 6.0-108.0(23.832±.6421) | 1.0-169.0(66.719±2.3782) |
| 3 | 1.0-12.0(8.989±.1047) | 5.0-128.0(29.948±2.2298) | 2.0-10.0(4.104±.2028) | 9.0-33.0(23.855±.3573) | 7.0-94.0(25.377±.6423) | 2.0-129.0(55.934±1.6144) |
| 4 | 2.0-18.0(12.695±.2058) | 9.0-172.0(39.582±2.5479) | 4.0-17.0(8.104±.3509) | 17.0-67.0(50.922±.4479) | 15.0-97.0(31.937±.4917) | 2.0-90.0(33.975±1.0111) |
| 5 | 2.0-30.0(24.194±.2305) | 6.0-200.0(54.320±2.4669) | 8.0-36.0(14.115±.6051) | 46.0-104.0(91.367±.8421) | 16.0-115.0(39.746±.6139) | 3.0-88.0(23.678±1.2852) |
| 6 | 9.0-68.0(36.558±.2861) | 2.0-287.0(73.392±2.9478) | 13.0-74.0(20.104±.7905) | 79.0-152.0(103.307±1.0846) | 17.0-144.0(50.278±.7798) | 0.0-83.0(16.318±1.4031) |
| 7 | 18.0-108.0(86.965±.6160) | 1.0-233.0(109.889±3.8937) | 28.0-91.0(40.542±1.2854) | 101.0-173.0(116.090±.6628) | 15.0-137.0(54.531±.7436) | 0.0-78.0(12.616±1.3932) |
| 8 | 13.0-103.0(86.763±.7580) | 3.0-171.0(94.882±3.0146) | 30.0-83.0(45.979±1.3935) | 84.0-156.0(100.952±.6710) | 13.0-135.0(44.436±.7853) | 0.0-80.0(14.781±1.4462) |
| 9 | 5.0-55.0(43.481±.4990) | 7.0-134.0(59.719±1.8961) | 13.0-44.0(22.365±.7692) | 67.0-135.0(106.416±.7008) | 10.0-110.0(40.657±.8210) | 0.0-87.0(20.690±1.6374) |
| 10 | 1.0-23.0(17.856±.2326) | 9.0-129.0(40.314±2.1665) | 6.0-19.0(10.708±.3726) | 17.0-76.0(55.705±.6199) | 15.0-111.0(36.994±.7631) | 1.0-117.0(35.698±1.7558) |
| 11 | 0.0-15.0(6.024±.0899) | 2.0-148.0(32.967±2.5797) | 1.0-7.0(3.427±.1706) | 3.0-30.0(11.060±.2077) | 12.0-144.0(38.006±.8446) | 0.0-162.0(57.731±1.6995) |
| 12 | 0.0-13.0(2.922±.0730) | 0.0-124.0(26.412±2.4601) | 0.0-4.0(1.031±.1009) | 1.0-7.0(2.060±.0911) | 10.0-193.0(36.379±.9145) | 0.0-169.0(76.236±2.5163) |

Table S6 Bioclimatic profiles of the six almond species based on the each month of the year (January is 1; December is 12) for water vapor pressure (kPa).

|  | *P. pedunculata* | *P. triloba* | *P. mongolica* | *P. tangutica* | *P. tenella* | *P. dulcis* |
| --- | --- | --- | --- | --- | --- | --- |
| 1 | 0.08-0.14(0.13±0.00) | 0.08-0.87(0.30±0.02) | 0.06-0.15(0.13±0.00) | 0.08-0.54(0.29±0.00) | 0.11-0.78(0.27±0.01) | 0.15-1.14(0.74±0.01) |
| 2 | 0.10-0.18(0.16±0.00) | 0.11-0.94(0.32±0.02) | 0.07-0.18(0.15±0.00) | 0.09-0.61(0.35±0.01) | 0.12-0.76(0.28±0.01) | 0.18-1.19(0.77±0.01) |
| 3 | 0.14-0.26(0.23±0.00) | 0.18-1.02(0.42±0.02) | 0.11-0.27(0.20±0.00) | 0.14-0.77(0.49±0.01) | 0.21-0.84(0.39±0.01) | 0.28-1.24(0.83±0.01) |
| 4 | 0.24-0.41(0.32±0.00) | 0.26-1.40(0.60±0.02) | 0.19-0.40(0.30±0.00) | 0.24-1.09(0.70±0.01) | 0.37-1.00(0.59±0.01) | 0.40-1.30(0.94±0.01) |
| 5 | 0.40-0.66(0.55±0.00) | 0.46-1.87(0.91±0.02) | 0.33-0.66(0.51±0.01) | 0.39-1.47(0.95±0.01) | 0.55-1.35(0.83±0.01) | 0.55-1.55(1.13±0.01) |
| 6 | 0.60-1.06(0.95±0.01) | 0.79-2.43(1.33±0.03) | 0.54-1.09(0.86±0.01) | 0.64-1.84(1.24±0.01) | 0.83-1.78(1.15±0.01) | 0.69-1.95(1.37±0.01) |
| 7 | 0.88-1.58(1.42±0.01) | 0.83-3.03(1.75±0.03) | 0.71-1.51(1.22±0.01) | 0.80-2.39(1.41±0.02) | 0.93-2.01(1.33±0.01) | 0.85-2.26(1.60±0.02) |
| 8 | 0.77-1.55(1.39±0.01) | 0.73-2.94(1.69±0.03) | 0.67-1.50(1.22±0.01) | 0.75-2.26(1.36±0.02) | 0.77-1.97(1.22±0.01) | 0.89-2.34(1.66±0.02) |
| 9 | 0.47-1.02(0.88±0.01) | 0.53-2.20(1.18±0.02) | 0.46-1.02(0.79±0.01) | 0.57-1.76(1.14±0.01) | 0.56-1.58(0.93±0.01) | 0.66-2.09(1.47±0.02) |
| 10 | 0.27-0.57(0.49±0.00) | 0.37-1.48(0.76±0.02) | 0.23-0.58(0.45±0.01) | 0.30-1.26(0.83±0.01) | 0.38-1.22(0.66±0.01) | 0.41-1.72(1.20±0.02) |
| 11 | 0.16-0.31(0.27±0.00) | 0.21-1.07(0.49±0.02) | 0.11-0.32(0.25±0.00) | 0.15-0.90(0.51±0.01) | 0.22-0.94(0.46±0.01) | 0.26-1.40(0.94±0.02) |
| 12 | 0.10-0.19(0.16±0.00) | 0.11-0.92(0.34±0.02) | 0.07-0.20(0.16±0.00) | 0.09-0.60(0.33±0.01) | 0.13-0.83(0.33±0.01) | 0.19-1.25(0.80±0.01) |

Table S7 Bioclimatic profiles of the six almond species based on the each month of the year (January is 1; December is 12) for solar radiation (kJ m^-2^ day^-1^).

|  | *P. pedunculata* | *P. triloba* | *P. mongolica* | *P. tangutica* | *P. tenella* | *P. dulcis* |
| --- | --- | --- | --- | --- | --- | --- |
| 1 | 4131.0-9603.0 (8921.614±39.3938) | 1095.0-10763.0 (6723.961±243.8019) | 8289.0-10045.0 (9529.354±36.0085) | 6524.0-9873.0 (8382.524±23.5000) | 993.0-6715.0 (4020.465±79.2512) | 1113.0-13045.0 (7824.198±166.3677) |
| 2 | 7932.0-12704.0 (12096.305±34.0168) | 3262.0-12897.0 (9433.118±258.4516) | 11654.0-13272.0 (12686.385±42.3415) | 8104.0-12719.0 (10241.145±35.7715) | 3130.0-10122.0 (7039.702±101.2886) | 3275.0-15616.0 (10598.913±178.5056) |
| 3 | 13253.0-16702.0 (16138.083±21.7539) | 7948.0-17282.0 (13296.105±241.9317) | 15753.0-16961.0 (16422.250±41.7318) | 10532.0-15804.0 (12399.060±37.4035) | 7669.0-13746.0 (11178.777±90.2651) | 8030.0-19825.0 (14640.983±160.0722) |
| 4 | 17031.0-20363.0 (19691.510±19.7443) | 12806.0-21622.0 (17111.935±184.3424) | 19027.0-20464.0 (19860.094±54.1626) | 13722.0-19446.0 (15143.627±41.3802) | 12743.0-18447.0 (15847.347±86.2156) | 13538.0-22811.0 (19005.426±139.5165) |
| 5 | 20095.0-23356.0 (22673.074±19.6548) | 15573.0-24085.0 (20541.516±153.3076) | 21921.0-23437.0 (22786.594±58.0853) | 15555.0-21635.0 (17103.241±49.2671) | 16971.0-22460.0 (20302.491±70.7036) | 17947.0-25352.0 (22781.231±122.7841) |
| 6 | 21107.0-24616.0 (23854.865±23.1274) | 16908.0-26022.0 (21581.203±151.3371) | 22904.0-24733.0 (24050.792±55.8285) | 15857.0-22215.0 (17673.536±61.1056) | 17619.0-24316.0 (22058.641±73.6475) | 19100.0-27797.0 (25405.595±139.6448) |
| 7 | 18907.0-23416.0 (22445.133±29.9953) | 15436.0-25808.0 (20364.529±133.7526) | 21955.0-23372.0 (22840.115±38.8286) | 15650.0-21158.0 (17607.741±60.0888) | 16824.0-23482.0 (21156.505±72.9083) | 18609.0-28074.0 (25351.789±155.2590) |
| 8 | 16248.0-21054.0 (20194.863±30.2613) | 14597.0-24133.0 (18268.843±159.7375) | 19882.0-21507.0 (20774.104±46.4791) | 15231.0-19546.0 (16738.663±43.8378) | 13586.0-21191.0 (18130.921±93.7087) | 14698.0-25699.0 (22808.624±185.9942) |
| 9 | 12645.0-18086.0 (17137.209±32.6364) | 8790.0-19995.0 (14813.712±239.0715) | 16733.0-18424.0 (17685.708±48.6129) | 11461.0-16773.0 (12861.614±35.8875) | 7953.0-16697.0 (13018.637±119.0730) | 9366.0-21739.0 (18343.021±217.7297) |
| 10 | 8648.0-14221.0 (13545.364±35.5683) | 4184.0-15322.0 (10713.549±268.5168) | 13523.0-14426.0 (13982.354±29.8817) | 8665.0-13664.0 (10138.235±33.2379) | 3897.0-11085.0 (7854.919±102.2733) | 4276.0-17067.0 (13368.579±220.4411) |
| 11 | 4976.0-10559.0 (9987.420±43.1205) | 1476.0-12144.0 (7283.660±252.3424) | 9412.0-10865.0 (10422.688±31.1319) | 7586.0-10664.0 (8907.602±23.6454) | 1237.0-7021.0 (4270.777±80.8472) | 1499.0-13019.0 (9310.161±197.2818) |
| 12 | 3164.0-9000.0 (8178.599±45.2353) | 668.0-10263.0 (5890.418±232.9916) | 7342.0-9159.0 (8609.448±36.2764) | 6157.0-8998.0 (7776.940±20.6141) | 523.0-5668.0 (3152.815±70.5843) | 677.0-11662.0 (7236.318±166.5039) |

Table S8 Bioclimatic profiles of the six almond species based on the each month of the year (January is 1; December is 12) for wind speed (m s^-1^).

|  | *P. pedunculata* | *P. triloba* | *P. mongolica* | *P. tangutica* | *P. tenella* | *P. dulcis* |
| --- | --- | --- | --- | --- | --- | --- |
| 1 | 1.4-4.0（2.649±0.0236） | 1.0-5.9（3.148±0.0781） | 2.1-3.6（2.658±0.0322） | 1.4-4.1（1.794±0.0298） | 1.2-5.9（3.159±0.0476） | 0.6-5.4（2.848±0.0637） |
| 2 | 1.5-4.9（2.893±0.0278） | 1.2-5.5（3.163±0.0663） | 2.4-3.9（2.965±0.0307） | 1.5-5.1（2.251±0.0401） | 1.4-5.5（3.264±0.0464） | 0.7-5.5（3.007±0.0639） |
| 3 | 2.1-5.7（3.373±0.0271） | 1.4-5.4（3.386±0.0629） | 2.7-4.3（3.379±0.0414） | 1.7-5.0（2.477±0.0371） | 1.8-5.4（3.195±0.0390） | 1.4-5.6（3.208±0.0542） |
| 4 | 2.8-6.2（3.854±0.0317） | 1.5-5.4（3.552±0.0631） | 3.0-4.8（3.853±0.0480） | 1.6-4.8（2.327±0.0374） | 1.9-4.8（3.490±0.0208） | 1.7-6.0（3.278±0.0513） |
| 5 | 2.7-5.9（3.851±0.0258） | 1.3-4.6（3.314±0.0531） | 2.7-4.6（3.876±0.0507） | 1.5-5.1（2.104±0.0383） | 1.7-4.6（3.353±0.0189） | 1.7-5.5（3.251±0.0467） |
| 6 | 2.3-4.7（3.258±0.0210） | 1.3-4.7（2.938±0.0458） | 2.6-3.9（3.388±0.0369） | 1.4-5.1（1.829±0.0372） | 1.6-4.7（3.022±0.0190） | 1.7-5.6（3.390±0.0484） |
| 7 | 1.8-4.0（2.877±0.0166） | 1.4-4.5（2.578±0.0468） | 2.2-3.8（3.114±0.0389） | 1.2-4.1（1.680±0.0275） | 1.6-4.5（2.853±0.0213） | 1.4-6.6（3.338±0.0535） |
| 8 | 1.8-3.9（2.636±0.0144） | 1.3-4.5（2.402±0.0479） | 2.1-3.6（2.985±0.0373） | 1.4-4.1（1.708±0.0283） | 1.4-4.5（2.796±0.0212） | 1.1-6.7（3.126±0.0535） |
| 9 | 2.0-4.4（2.651±0.0244） | 1.2-5.4（2.556±0.0558） | 2.0-3.6（2.833±0.0383） | 1.3-4.5（1.702±0.0328） | 1.4-5.4（2.936±0.0242） | 1.2-5.4（2.975±0.0483） |
| 10 | 1.9-4.7（2.891±0.0281） | 1.2-5.5（2.812±0.0599） | 2.2-3.6（2.902±0.0400） | 1.2-4.6（1.845±0.0354） | 1.3-5.5（3.175±0.0286） | 1.1-5.0（2.741±0.0513） |
| 11 | 1.7-5.0（2.812±0.0292） | 1.1-5.8（3.005±0.0698） | 2.3-3.8（2.829±0.0378） | 1.3-4.1（1.723±0.0278） | 1.3-5.8（3.202±0.0361） | 0.8-5.4（2.631±0.0623） |
| 12 | 1.5-4.3（2.619±0.0276） | 1.1-5.7（3.005±0.0736） | 2.1-3.7（2.644±0.0312） | 1.1-4.2（1.477±0.0313） | 1.1-5.7（3.130±0.0459） | 0.7-5.3（2.767±0.0648） |

Table S9 Accession numbers of chloroplast genomes used for phylogenetic analyses.

| Family | Genus | Taxon | Genbank No. | Source |
| --- | --- | --- | --- | --- |
| Amygdaleae | *Prunus* | *Prunus dulcis* | MH727486 | Scientific Reports 10, 10137 (2020) |
| Amygdaleae | *Prunus* | *Prunus pedunculata* | MG869261 | Conservation Genetics Resources 11, 419-421 (2018a) |
| Amygdaleae | *Prunus* | *Prunus triloba* | MH748555 | Scientific Reports 10, 10137 (2020) |
| Amygdaleae | *Prunus* | *Prunus mongolica* | MH727485 | Scientific Reports 10, 10137 (2020) |
| Amygdaleae | *Prunus* | *Prunus tangutica* | MH744156 | Scientific Reports 10, 10137 (2020) |
| Amygdaleae | *Prunus* | *Prunus tenella* | MH727487 | Scientific Reports 10, 10137 (2020) |
