# Supplementary material for: Current Situation, Global Potential Distribution and Evolution of Six Almond Species in China
Source: Front Plant Sci. 2021 Apr 23;12:619883. doi: 10.3389/fpls.2021.619883 (PMC8102835; doi:10.3389/fpls.2021.619883)
Supplement: Supplementary file 6 [file Data_Sheet_2.docx]

**Figure S1** (A) Score plot of principal components for 19 bioclimatic parameters from the six almond species; (B) Loadings plot of principal components for pc1 from the six almond species; (C) Loadings plot of principal components for pc2 from the six almond species.

**Figure S2** The average omission and predicted area for the six almond species.

**Figure S3** Receiver operating characteristics curve (ROC) for training and test data with the area under the ROC curve (AUC).

**Figure S4** The Jackknife test for evaluating the relative importance of environmental variables for each almond species.
